# Multisensor Localization and Risk-Aware Navigation for Underwater Robots in Turbid and Confined Inland Waters: A Review

**DOI:** 10.3390/s26185822

**Published:** 2026-09-14

**Authors:** Ruifan Tang, Qianrun Zang, Yu Zhang, Yapeng Wu, Jiewen Yang, Zhong Tang

**Affiliations:** College of Agricultural Engineering, Jiangsu University, Zhenjiang 212013, China; 3252502021@stmail.ujs.edu.cn (R.T.);

**Keywords:** inland waters, underwater robots, multisensor fusion, underwater localization, sensor degradation, localization integrity, risk-aware path planning

## Abstract

Inland waters such as lakes, reservoirs, rivers, fish ponds, and confined hydraulic structures impose turbidity, shallow-water acoustic multipath, dense boundaries, dynamic biological interference, and limited communication on underwater robots. These coupled constraints can simultaneously degrade sensing, localization, mapping, planning, and control. This structured narrative review synthesizes peer-reviewed English-language evidence on underwater navigation, with emphasis on multisensor fusion localization and the transfer of localization uncertainty into risk-aware planning. Visual, acoustic, inertial, velocity, depth, and external positioning measurements are compared by complementarity, observability, degradation modes, and integration cost. Filtering, sliding-window optimization, factor graphs, estimator switching, and hybrid model- and data-driven approaches are evaluated according to accuracy, real-time performance, robustness, and localization credibility. The review examines how covariance, sensing quality, collision probability, energy, platform dynamics, and task requirements affect maps, path costs, safety margins, trajectory tracking, and degraded operation. Evidence from simulation, public datasets, hardware-in-the-loop tests, tanks, and real waters is synthesized conditionally because study platforms, environments, ground truth, and metrics are heterogeneous. Reliable inland-water autonomy requires diagnosable heterogeneous sensing, integrity-aware localization, and coordinated feedback among localization, planning, and control. Mission-level evaluation should consider data validity, fault recovery, and safe completion rather than average localization error or shortest path alone.

## 1. Introduction

### 1.1. Application Background and Research Significance of Underwater Robots in Inland Water Bodies

Inland water bodies support water resource regulation, ecological protection, water supply, aquaculture, and hydraulic infrastructure. These functions create recurring needs for water quality monitoring, underwater mapping, structural inspection, aquaculture facility inspection, and biological observation. Manual sampling, diver inspection, fixed stations, and tethered robots are limited by safety, coverage, repeatability, or operating duration. Autonomous underwater robots can carry visual, acoustic, and environmental sensors while attaching spatial and temporal references to the acquired data [1,2].

Navigation determines whether inspection observations can be associated reliably with their acquisition locations. Localization bias weakens repeated water quality comparisons, while pose drift impairs image or sonar registration during structural inspection. Unstable near-wall motion can also cause missed coverage, collision, or disturbance to aquatic organisms. Localization, planning, and control must therefore operate as a coordinated loop that supports data registration, task coverage, safety, and energy assessment.

Inland operations commonly favor small platforms, low-cost deployment, convenient maintenance, and frequent reuse. Sustained accuracy must therefore be achieved under restricted payload, power, computation, and budget. Multisensor fusion and uncertainty-aware planning provide a basis for maintaining localization continuity and generating executable inspection paths under these constraints. Reviewing their applicability, failure boundaries, and integration requirements can support platform configuration and engineering validation.

### 1.2. Environmental Constraints and Core Problems of Autonomous Navigation in Inland Water Bodies

**Figure 1:**
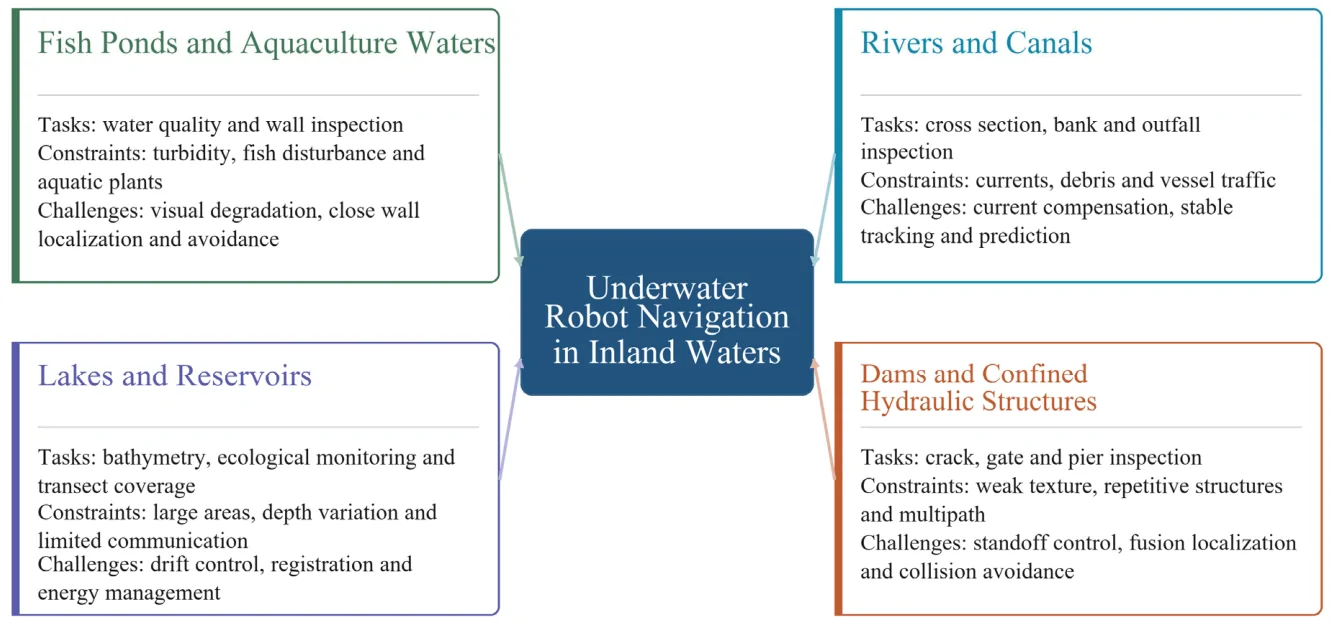
Relationship among inland water scenarios, inspection tasks, and navigation challenges. Source: Created by the authors; no external artwork is reproduced.

Inland waters are not reduced-scale marine environments. Figure 1 links representative settings and inspection objectives to their principal navigation challenges.

Turbidity, shallow-water acoustic multipath, repetitive boundaries, currents, biological motion, and restricted communication can degrade several sensing modalities at once. Because Global Navigation Satellite System (GNSS) signals do not propagate effectively underwater, localization usually combines an inertial measurement unit (IMU), depth and heading sensors, a Doppler Velocity Log (DVL), cameras, sonar, or external acoustic references [3,4,5]. Section 2 provides the detailed scenario and failure analysis.

This review addresses three coupled problems: selecting complementary sensors under resource constraints, maintaining credible localization during degradation, and converting uncertain state estimates into safe, observable, energy-aware, and executable paths.

### 1.3. Advances in Underwater Sensing, Multisensor Localization, and Risk-Aware Planning

Underwater navigation has progressed from aided inertial dead reckoning to tightly coupled estimation and simultaneous localization and mapping (SLAM) [3,4,5,6,7]. Sliding-window and graph-based methods improve heterogeneous measurement use, but remain limited by visibility, acoustic propagation, calibration, and computation.

Multimodal systems combine visual, acoustic, inertial, velocity, depth, and external observations to preserve observability during partial degradation [8,9,10]. Evaluation has therefore expanded from average error to outlier rejection, fault isolation, integrity monitoring, and whether the estimate remains usable for downstream decisions.

Planning methods include graph and incremental search, sampling, intelligent optimization, and learning [11,12]. Inland missions require these methods to consider energy, currents, dynamic obstacles, coverage, sensor observability, vehicle dynamics, and onboard resources.

Risk-aware planning introduces uncertainty or failure probability into costs and constraints [13,14]. Localization covariance, sensor health, and map confidence can then influence route selection, safety margins, replanning, and active relocalization.

**Figure 2:**
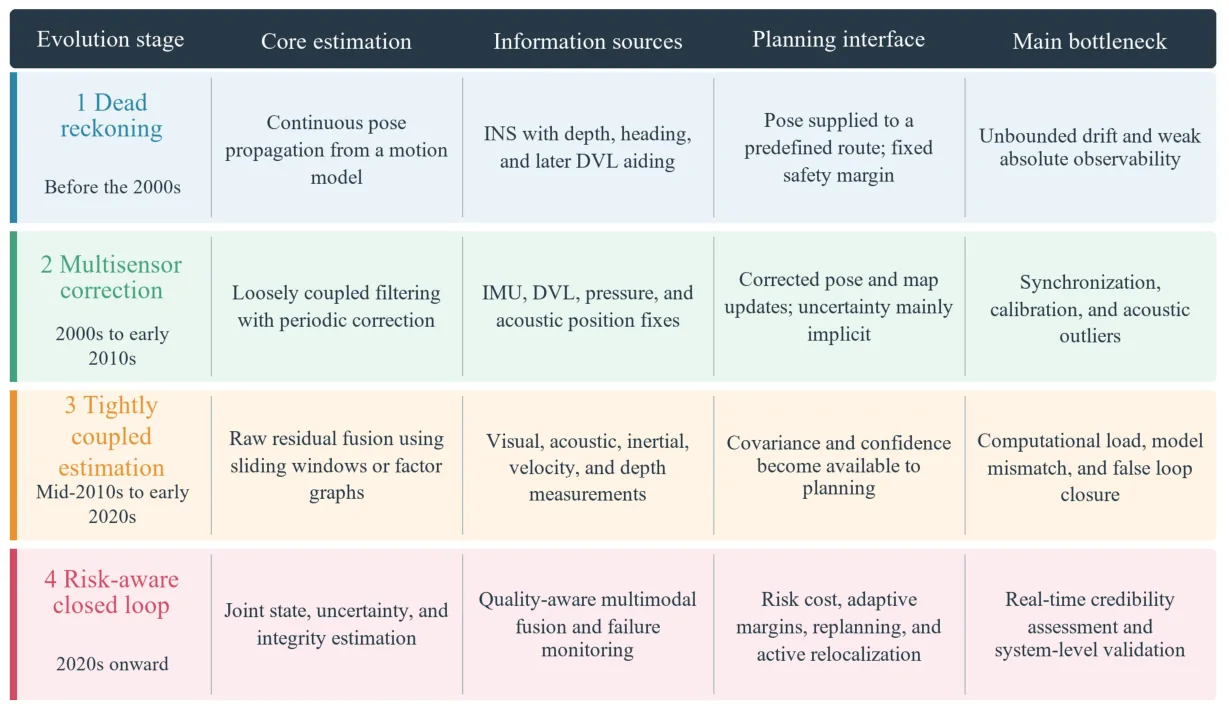
Technology development roadmap for multisensor fusion localization and risk-aware navigation of underwater robots. Source: Redrawn and synthesized by the authors from the technological concepts reported in Refs. [8,14,15,16]; no source artwork is reproduced. The periods indicate dominant research emphases rather than exclusive chronological boundaries.

Figure 2 summarizes this development from dead reckoning and multisensor correction to tightly coupled estimation and risk-aware closed-loop navigation [8,14,15,16].

Existing reviews establish foundations in integrated navigation [3,4,5], underwater SLAM [6,7], path planning [11,12], and platform applications [1,2,17,18,19]. Their differing environments and evaluation criteria provide limited guidance for configuring a complete inland-water inspection system.

Three gaps motivate this review: coupled inland-water degradation is not consistently linked to sensor selection; localization credibility is rarely traced through planning and control; and heterogeneous experiments impede comparison of real-time feasibility and engineering maturity.

This review therefore compares sensing, fusion, confidence, planning, control, platform integration, and validation within a common sensing, localization, planning, and control (SLPC) framework. Table 1 positions this scope relative to existing reviews.

This review contributes an inland-water-specific SLPC failure chain, treats localization credibility as the interface between sensing and risk-aware navigation, compares methods under algorithmic and implementation constraints, and organizes evidence by validation maturity to support task-conditioned system configuration.

Figure 2 presents a technology roadmap rather than a performance ranking. Its four stages use consistent dimensions: estimation structure, information sources, planning interface, and principal bottleneck. The periods denote dominant emphases because the approaches coexist.

Aided dead reckoning used depth, heading, velocity, and acoustic updates to slow drift. Tight coupling later introduced raw measurement residuals and covariance transfer. Current risk-aware systems seek to pass pose credibility to planning so that costs, margins, replanning, and active relocalization respond to degradation [8,14,15,16].

The bottleneck has consequently shifted from drift suppression to credible real-time decisions. Synchronization error, calibration drift, outliers, false loop closures, and delay can make a precise-looking estimate unsuitable for planning; confidence validity must therefore be evaluated with state accuracy.

### 1.4. Review Method and Sensor System Analytical Framework

This structured narrative review uses database searching and backward and forward citation tracking. It is not a systematic review or meta-analysis. The evidence covers English-language research on underwater sensing, localization, mapping, planning, control, integration, and validation. Non-inland studies were retained only when their principles and operating assumptions were technically transferable.

The principal discovery sources were Web of Science Core Collection, Scopus, IEEE Xplore, ScienceDirect, and SpringerLink; Google Scholar and citation tracking supplemented retrieval. The search was updated through 23 July 2026. Because this is a structured narrative review, PRISMA-style screening counts are not claimed.

Search blocks covered underwater platforms, inland and confined waters, multisensor localization and SLAM, and planning, obstacle avoidance, inspection, and control. Platform terms were combined with at least one environment, localization, planning, or task block, with syntax adapted to each database.

Eligible English-language studies addressed an underwater platform or a technically defensible transfer method and provided sufficient information on sensing, localization, mapping, planning, control, integration, or validation. Peer-reviewed journals were prioritized; other English sources were retained only for necessary foundational or engineering evidence.

Duplicate, weakly related, bibliographically untraceable, and methodologically insufficient records were excluded. Non-underwater evidence was retained only when its transfer pathway was explicit. Marine evidence was interpreted within its environmental, platform, risk, and validation boundaries.

Figure and Table Copyright Statement. All reused or adapted source artwork in this manuscript is supported by archived CC BY 4.0 evidence. Author-created and independently redrawn figures reproduce no source artwork. Each figure caption identifies its source, adaptation status, and license where applicable.

Extraction fields covered platform, environment, task, sensors, calibration, synchronization, fusion, mapping, planning, control, computing, ground truth, performance, and failure modes. Evidence was classified as repeated real-water, short-field, tank- or hardware-in-the-loop, public-dataset, or simulation validation.

The synthesis uses thematic grouping and conditional technical comparison rather than statistical pooling. Values are retained only with their source conditions. The evidence base is therefore interpreted by scenario, platform, sensing configuration, ground truth, and validation level.

### 1.5. Scope, Sensor-Centric Research Questions, and Article Organization

This review covers lakes, reservoirs, rivers, canals, urban waterways, fish ponds, aquaculture waters, artificial storage basins, and confined hydraulic facilities. It primarily considers autonomous underwater vehicles (AUVs), remotely operated vehicles (ROVs) with autonomous navigation functions, and underwater platforms in cooperative surface-underwater systems. The technical scope includes platform integration, multisensor localization, localization credibility, mapping, risk-aware planning, local obstacle avoidance, trajectory control, and system validation.

Four questions guide the review. How do inland-water environments and inspection tasks constrain sensing, localization, planning, and control? Under what conditions are inertial, depth, velocity, visual, acoustic, and external measurements complementary? How should localization error, covariance, and degradation propagate to maps, paths, control, and inspection quality? How should platforms, sensors, and algorithms be configured to balance cost, accuracy, robustness, real-time performance, and engineering maturity?

**Figure 3:**
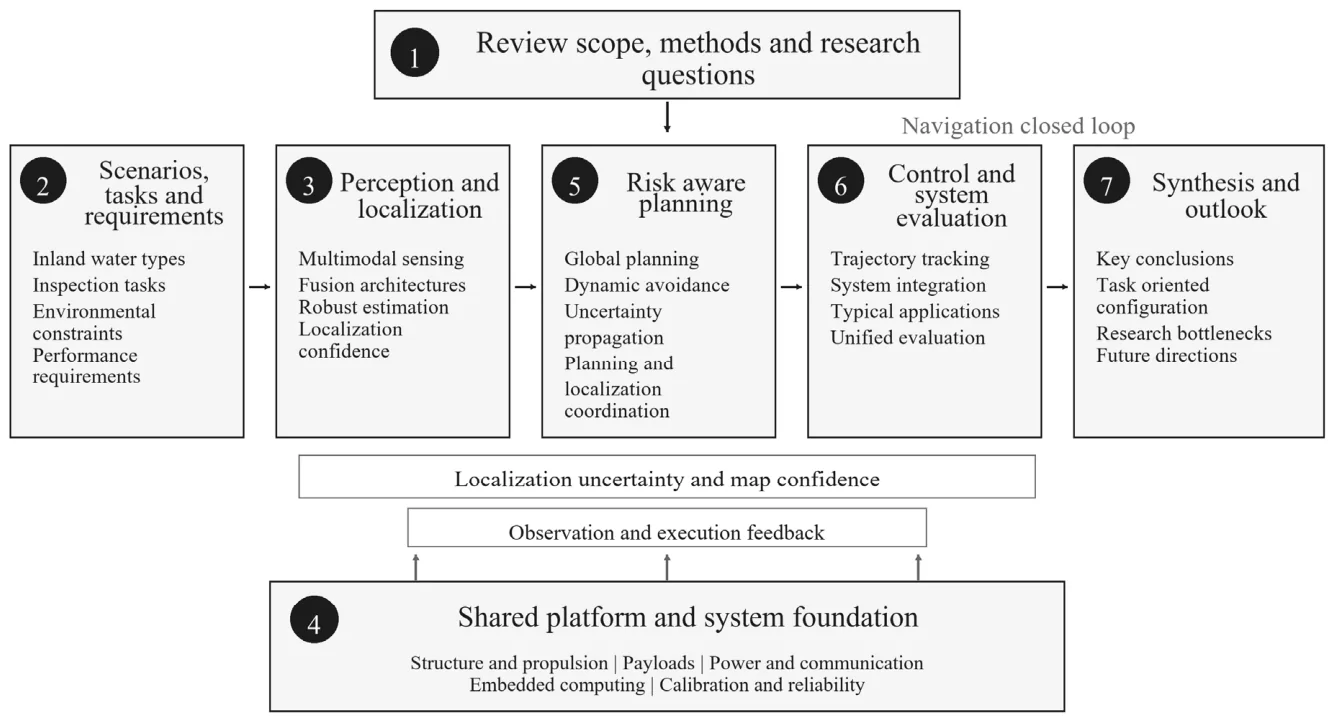
Comprehensive analytical framework and organization of this review. Source: Compiled and redrawn by the authors; no external artwork is reproduced.

Section 2, Section 3, Section 4, Section 5 and Section 6 follow the SLPC chain from scenarios and sensing through platform integration, planning, control, and validation. Section 7 synthesizes limitations and research directions, and Section 8 concludes. Figure 3 shows this analytical organization.

## 2. Inspection Scenarios, Task Requirements, and Navigation System Constraints for Inland Water Bodies

### 2.1. Types of Inland Water Bodies and Their Navigational Environmental Characteristics

This section places the detailed scenario taxonomy omitted from Section 1 into four groups: lakes and reservoirs, rivers and canals, fish ponds and aquaculture waters, and confined hydraulic facilities. They are compared by scale, flow, boundaries, visibility, acoustic propagation, observability, and safety margin.

Lakes and reservoirs combine open-water surveys with constrained near-dam inspection. Long range and sparse communication increase inertial drift and energy demand, whereas slopes, gates, outlets, and sediments increase near-wall localization and collision avoidance requirements. Mission data remain useful only when samples, depths, and timestamps are registered reliably [20], and pose uncertainty directly affects bathymetric swath alignment [21].

Rivers and canals combine directional flow, narrow corridors, and rapidly changing obstacles. Flow shear affects tracking and energy, while repetitive banks weaken longitudinal visual or sonar observability. Incremental maps, online replanning, current compensation, and velocity, depth, heading, or acoustic constraints are therefore required [3,4,5].

Fish ponds and aquaculture waters favor compact low-cost vehicles but combine suspended matter, plants, bubbles, regular walls, and dynamic fish. These factors degrade optical and acoustic observations and increase near-wall risk. Geometric priors can assist localization [22], but methods transferred from offshore cages require revalidation for pond scale, flow, occlusion, and safety distance.

Confined hydraulic facilities contain narrow passages, repetitive structures, and strong reflections. Acoustic multipath, weak texture, and limited maneuverability reduce single-sensor reliability, whereas structural priors, markers, and multisensor fusion can improve observability [15]. Inspection also requires platform motion, standoff, illumination, and payload field of view to be designed together [23].

Adjacent surface monitoring evidence helps define synchronization and degradation tests for fish ponds [24,25], but cannot substitute for underwater platform validation.

### 2.2. Typical Inspection and Detection Tasks

Inland tasks comprise water quality and ecological monitoring, bathymetric and environmental mapping, structural inspection, and aquaculture observation. Their outputs are georeferenced parameter fields, maps, defect records, and biological or facility status. Navigation metrics should therefore be derived from the required data product, not average position error alone [26,27].

Water quality monitoring includes fixed stations, profiles, transects, and grids. These modes emphasize repeat arrival, depth, coverage spacing, speed, timing, and limited biological disturbance. Field applications show that localization quality, biofouling control, and data interpretation remain central to sustained three-dimensional monitoring [20].

Bathymetric and environmental mapping uses visual or acoustic swaths to reconstruct terrain, sediment, and obstacles. Pose error appears as layer offsets, strip misalignment, and scale distortion; evaluation should therefore include overlap registration, map consistency, and loop closure effects [21].

Structural inspection requires near-wall distance, low-speed stability, surface coverage, and repeatable viewing geometry. Because payload quality, attitude, and local pose error are coupled [22,23], defect records should retain position confidence, standoff, and image or sonar quality.

Aquaculture observation includes fish distribution and behavior, mortality, net damage, and equipment anomalies. Dynamic fish are both targets and obstacles; planning must balance coverage, observation angle, disturbance, collision risk, and rejection of moving localization features [1,28,29,30].

**Figure 4:**
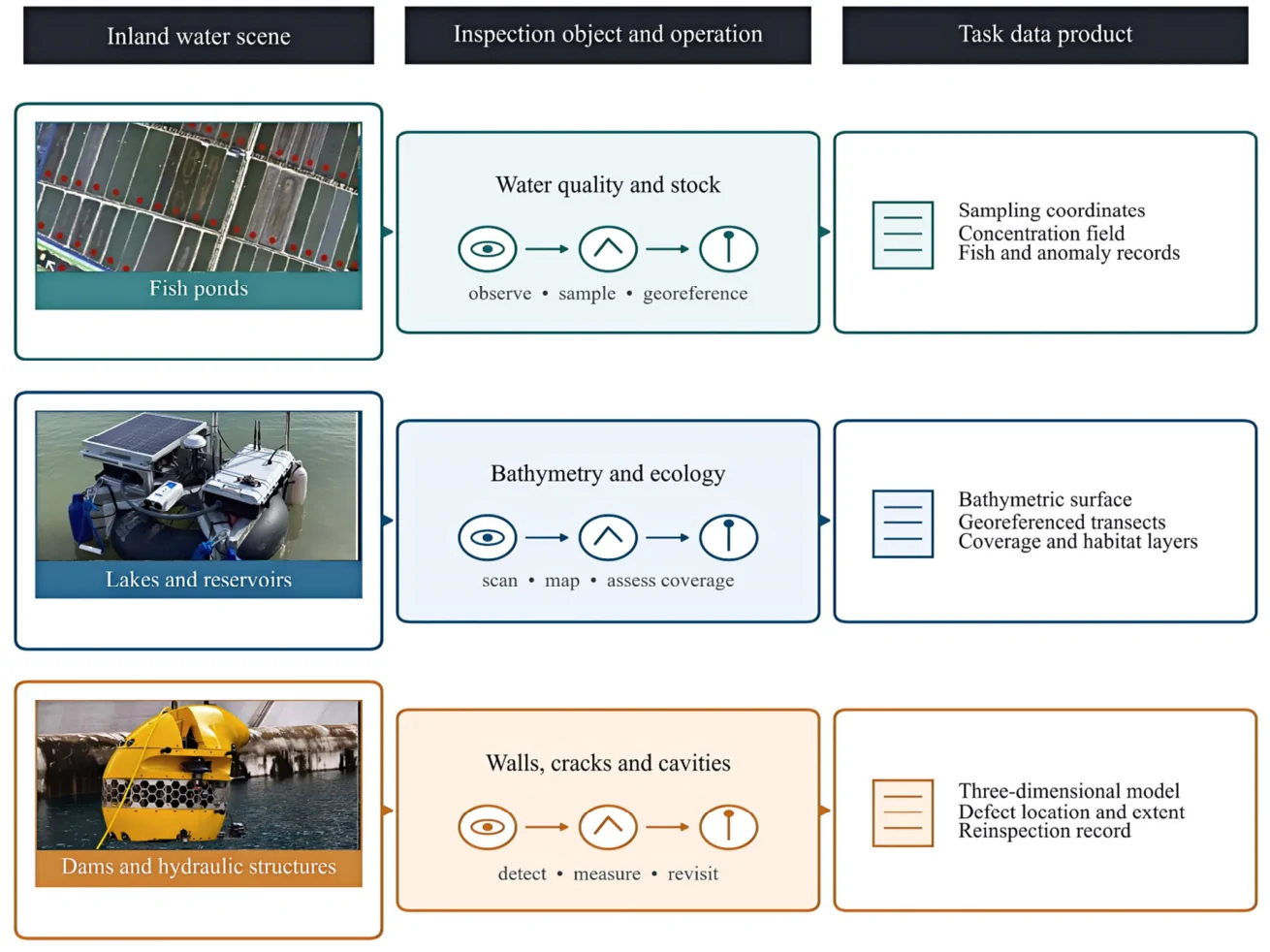
Representative inland water environments, inspection objects, and task data products. The three left-column photographs were cropped and adapted from Ma et al. [31], Figure 1; Sotelo-Torres et al. [32], Figure 2; and Zhao et al. [33], Figure 1, respectively, under the Creative Commons Attribution 4.0 International License (CC BY 4.0). The remaining schematic elements were created by the authors. The figure presents selected environments for visual clarity; the broader classification, including rivers, canals, and urban waterways, is provided in Table 2. The red dots and arrows are scene indicators added to improve visual readability; they do not represent quantitative annotations or independent measurements.

Figure 4 presents three representative scenario links: pond water quality monitoring, lake and reservoir bathymetric surveying, and dam structure inspection. The left-column photographs are cropped from open access studies of pond aquaculture [31], an autonomous bathymetric survey platform [32], and a hydropower dam inspection robot [33], respectively. The figure is a compact visual illustration rather than an exhaustive classification; Table 2 additionally covers rivers, canals, urban waterways, and other inland water environments. Across these cases, the robot must preserve usable observation geometry and produce spatially referenced, comparable, and reviewable data products.

Related monitoring studies reinforce the need for continuous, spatially referenced data products [34,35,36,37,38]. Their transferable contribution is the shared spatiotemporal reference between payload data, trajectory, and detection result, rather than the domain-specific operating mechanism.

**Table 2:** Inland water types, autonomous inspection tasks, main disturbances, and navigation challenges. Data source: the scenario and task synthesis in Section 2.1, Section 2.2, Section 2.3 and Section 2.4 and Refs. [33,39]. The entries describe condition-dependent navigation requirements rather than universal environmental values.

Water Body Type	Typical Autonomous Inspection Task	Main Environmental and System Disturbances	Navigation Challenges and Key Metrics
Lakes and reservoirs	Water quality profiling, ecological monitoring, bathymetry, and near-dam inspection	Large scale, varying water depth, intermittent communication, and dense near-dam structures	Long-voyage drift, endurance, swath registration, and near-wall safety
Rivers, canals, and urban waterways	Cross-section monitoring, riverbed mapping, and inspection of bridge piers and revetments	Directional currents, narrow channels, vessels, and floating objects	Flow-field compensation, dynamic replanning, return energy, and observability along the route
Fish ponds and aquaculture waters	Water quality sampling, fish school observation, and inspection of pond walls, pond bottoms, and facilities	Turbidity, bubbles, aquatic vegetation, regular boundaries, and dynamic fish schools	Low-cost fusion, near-wall distance keeping, dynamic obstacle avoidance, and low-disturbance navigation
Artificial pools and enclosed hydraulic facilities	Inspection of gates, dam interiors, reservoirs, and confined components	Weak texture, repetitive structures, acoustic multipath, and spatial constraints	Use of structural priors, stable imaging, collision margin, and failure degradation

### 2.3. Autonomous Navigation Task Modes

Inspection tasks reduce to four navigation modes: point or transect inspection, area coverage and search, boundary following, and dynamic avoidance with safe return. They emphasize, respectively, arrival and cross-track error, omissions and repetition, standoff and attitude, and online risk response.

Point and transect missions revisit sampling stations or prescribed routes. Evaluation should include arrival, cross-track and repeat-track errors, station keeping, and localization availability, including recovery after brief sensor loss.

Coverage and search missions use decomposition, sweeping, graph search, or optimization while respecting turning radius, sensor swath, current, energy, and drift [40]. Coverage, repetition, missed detections, information gain, and energy per area should be reported together.

Boundary inspection maintains standoff and viewing angle along walls, structures, pipes, or nets. Boundary loss, abrupt curvature, and occlusion can interrupt tracking [41], and thruster limits mean that geometric feasibility alone does not ensure executable motion [42].

Dynamic avoidance and safe return address organisms, floating objects, sudden flow, degraded localization, and low energy. Responses should use predicted motion, braking distance, localization confidence, communication, and energy rather than instantaneous clearance alone.

### 2.4. Operating Mechanisms and Failure Modes of Environmental Constraints

**Figure 5:**
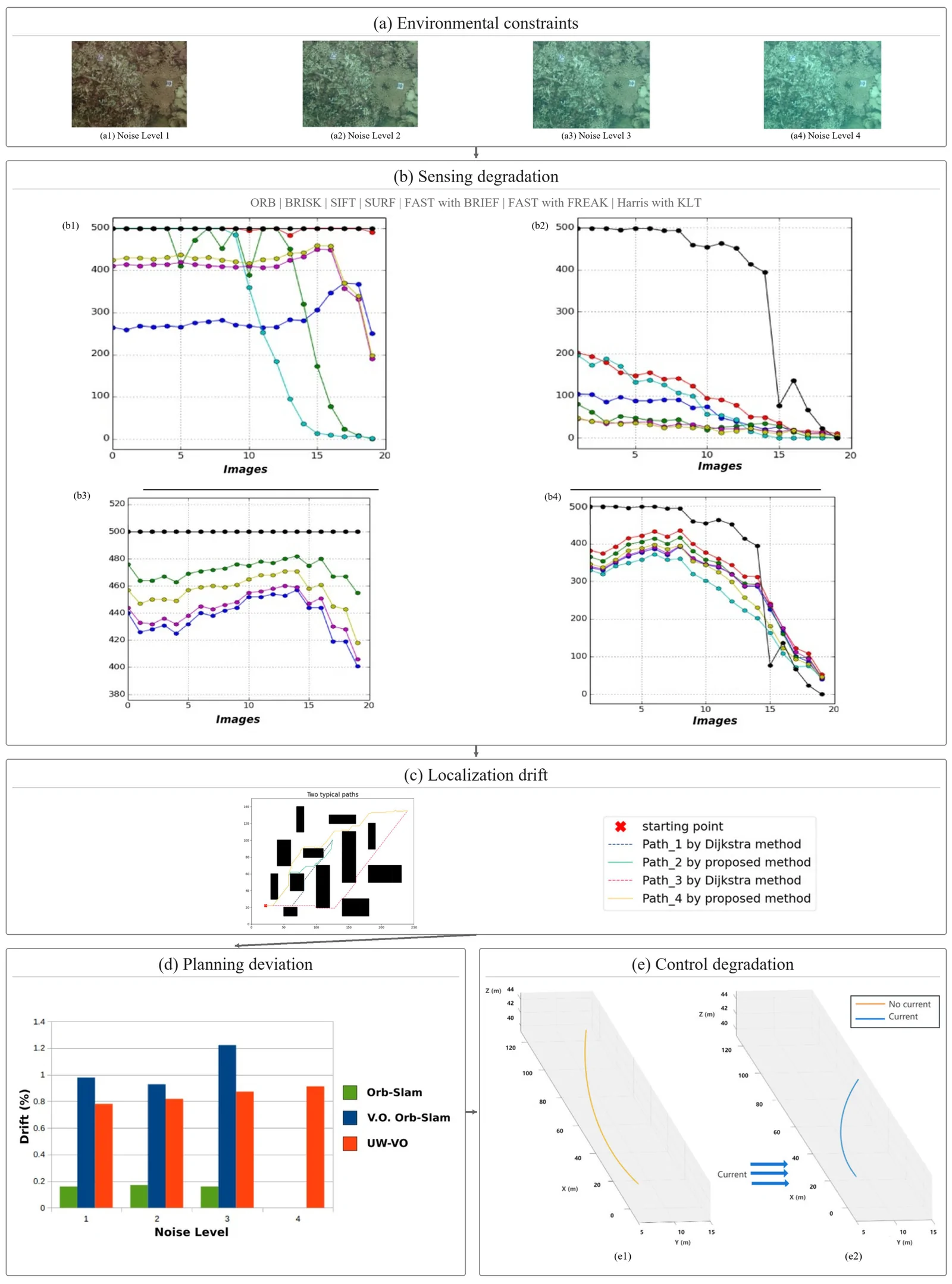
The four source views in panel (**a**) show increasing environmental noise levels, the plots in panel (**b**) show sensing degradation under alternative feature-detection or matching conditions, panel (**c**) shows localization drift and alternative paths, panel (**d**) shows planning deviation at increasing noise levels, and panel (**e**) shows control degradation under no-current and current conditions, with (**e1**,**e2**) denoting the two conditions. Within the source panels, colored curves, paths, bars, and trajectories retain the meanings assigned by the corresponding source legends. The internal labels follow the visual reading order: (**a1**–**a4**) for the four environmental views, (**b1**–**b4**) for the four sensing-degradation plots, and (**e1**,**e2**) for the two control conditions. Source: Panels (**a**–**c**) were adapted from Ref. [43], Figures 6, 3, and 7; panel (**d**) from Ref. [19], Figure 6; and panel (**e**) from Ref. [45], Figure 9. All source articles are licensed under CC BY 4.0. The panels were cropped and rearranged by the authors.

Inland-water constraints form a failure chain from physical conditions through observations, state estimation, maps, planning, and control. Figure 5 identifies where mitigation should act instead of treating turbidity, complexity, and dynamics as undifferentiated problems.

Turbidity, weak illumination, scattering, and repetitive structures reduce contrast, feature count, and association reliability, causing visual odometry drift or false loop closure [34,43]. Enhancement should therefore be judged by inlier ratio, tracking duration, and pose improvement rather than visual appearance.

Shallow-water multipath and nonstationary scattering can corrupt sonar range and association. DVL bottom lock can fail because of range, reflectivity, attitude, or occlusion, after which inertial error grows. Alternative velocity estimates can bridge short gaps, but still require anomaly detection and uncertainty assessment [35].

Quantitative evidence is configuration-specific. A Tokyo Bay ship test used a 100 Hz IMU, 1 Hz DVL, and 5 Hz GNSS reference for 1 h. The maximum yaw difference reached about 15 deg, while fused velocity had a 0.09 m/s standard deviation and 0.53 m/s maximum error [36]. UX-1 reported 500 m multibeam and 75 m mechanical sonar ranges and a 9 fps structured light system [37]. These values are test and instrument specifications, not universal inland-water sensing limits.

Currents and dynamic organisms affect motion and sensing simultaneously. Persistent flow biases motion prediction, vortices perturb attitude, and fish or floating objects may become false landmarks and hazards. Disturbance estimation, dynamic feature rejection, and uncertainty-dependent safety margins are therefore required [38].

**Figure 6:**
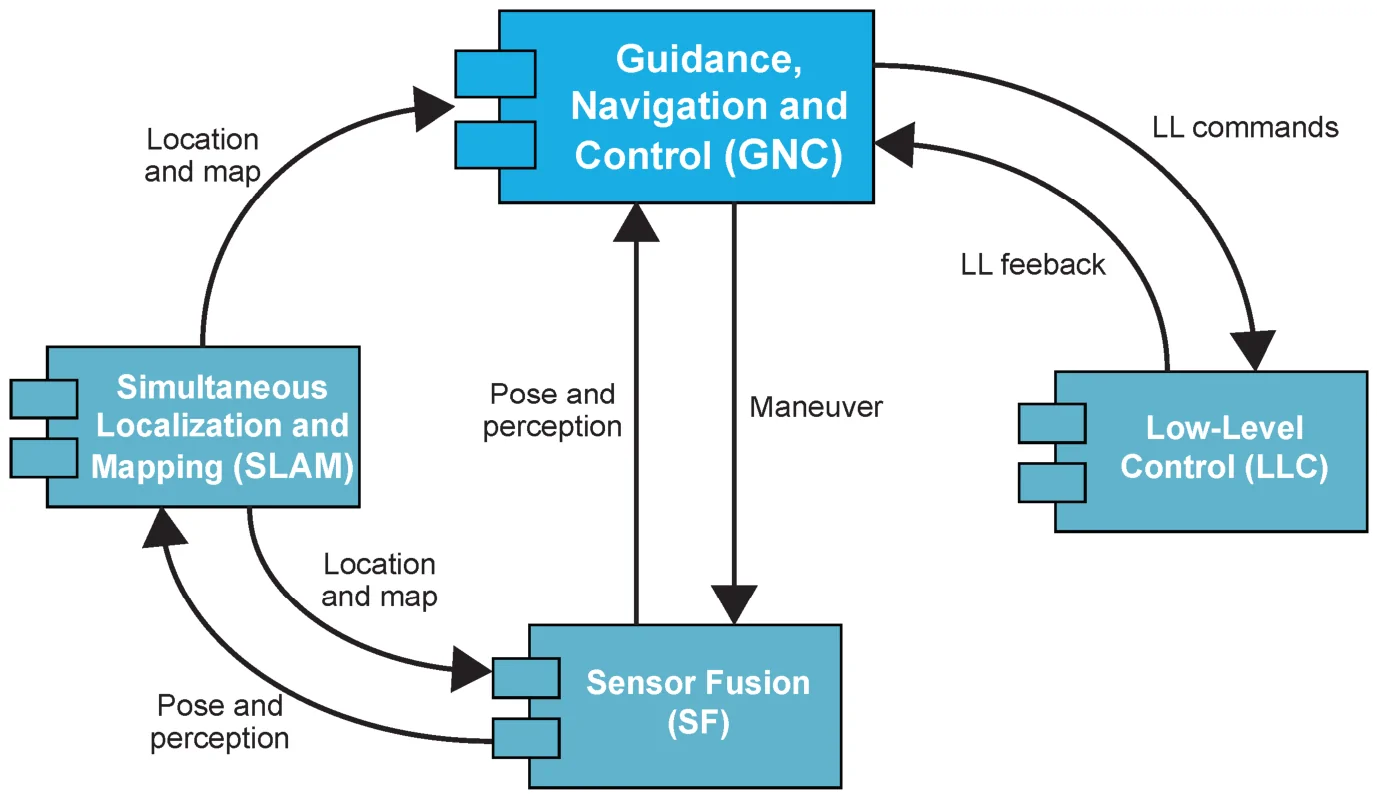
Closed-loop architecture of simultaneous localization and mapping, sensor fusion, guidance, navigation, and control for underwater robots. Source: Reproduced from Ref. [37], Figure 4, under CC BY 4.0. GNC denotes guidance, navigation, and control; LLC denotes low-level control. The arrows indicate the information and control-flow directions shown in the source figure.

Communication, computation, energy, and payload limits create system-level failures. Low-bandwidth intermittent links constrain supervision [44], while cost, space, clocks, and power restrict redundancy. Low-cost navigation therefore requires calibration, health monitoring, and explicit degraded operation [45]. Figure 6 traces these constraints into localization, planning, and control consequences.

### 2.5. Navigation Performance Metrics and the Sensing, Localization, Planning, and Control (SLPC) Architecture

The sensing, localization, planning, and control (SLPC) architecture converts inspection requirements into a closed set of measurable interfaces. In the confined environment system shown in Figure 6, SLAM and sensor fusion supply pose and maps to navigation, guidance, and control; low-level control executes thruster commands and returns vehicle state [37]. Mission management supervises degradation and safety. Table 2 provides the broader classification used throughout this review, including rivers, canals, urban waterways, and other environments not individually illustrated in Figure 4. Table 3 summarizes the stage-specific performance metrics.

Sensing metrics include range and field of view, missed and false detections, latency, tracking duration, update rate, and map consistency. Outputs should include confidence, timestamps, and sensor health so that downstream modules can recognize turbidity, weak texture, multipath, occlusion, and biological interference.

Localization evaluation should combine absolute and relative trajectory error, heading and depth error, repeat arrival, availability, consistency, failure detection, and relocalization. Mean error describes nominal accuracy, whereas high-percentile error and integrity measures define safety relevance. Mapping tasks should also report registration error [3,10].

Planning metrics include path cost, computation and replanning time, clearance, collision risk, curvature, success, and energy margin. Pose and map uncertainty should influence obstacle inflation, speed, information gain, or relocalization rather than being treated as certain [12,40].

Control metrics include tracking error, settling and recovery, smoothness, saturation, and energy. Persistent error or reduced actuation should feed back to model correction and replanning; unsafe localization, energy, or propulsion states should trigger deceleration, relocalization, return, or surfacing [41,44].

Table 3 organizes the recommended metrics by sensing, localization, planning, control, and mission level, and identifies the meaning and applicable task of each metric.

Figure 6 closes the SLPC loop: sensing quality conditions observability, localization uncertainty sets planning margins, planned motion must remain trackable, and execution feedback updates estimation and replanning [37]. Mission-level coverage, repeatability, time, energy, and success cannot be replaced by average performance of one module.

The following chapters apply this chain to credible localization, platform constraints, uncertainty-aware planning, control, and closed-loop validation.

## 3. Multimodal Environmental Sensing and Multisensor Fusion Localization Methods

Section 2 summarized the environmental constraints of inland water bodies as a chainwise transmission among sensing, localization, planning, and control, in which a reliable pose is the common basis for subsequent map updates and risk decision-making. On this basis, this chapter starts from navigation information sources and their complementary relationships, and then reviews visual and acoustic front ends, fusion levels, typical sensor combinations, and robust mechanisms under nonideal conditions. This chapter compares the constraints provided by each method and the conditions under which those constraints remain valid. It also identifies their failure boundaries. The central question is how a system can progress from outputting a pose to outputting a trustworthy pose for planning.

### 3.1. Navigation Information Sources and Complementary Mechanisms

The technical question addressed in this section is which information sources can maintain an observable and credible navigation state when no single sensor remains reliable throughout an inland-water mission. The relevant sources should be compared under six common dimensions: estimation accuracy, robustness to environmental change, computational cost, environmental adaptability, characteristic failure mode, and deployment condition. An IMU offers high-rate propagation at low computational cost, but integrated bias produces unbounded drift. A pressure sensor supplies a stable vertical constraint but does not correct horizontal error. A DVL suppresses inertial drift only while valid bottom-lock or water-lock measurements are available. These sensors therefore form a continuous navigation backbone rather than a complete positioning solution [20,46,47,48,49].

Vision provides dense texture, local geometry, semantic cues, and relative motion at comparatively low hardware cost. Monocular vision has a scale-observability limitation, whereas stereo, visual–inertial, and pressure-assisted schemes improve scale or depth estimation. Its accuracy can be high in clear, textured, and adequately illuminated water, but robustness decreases under turbidity, color attenuation, weak texture, repetitive boundaries, and moving organisms. Dataset evidence shows that these degradations first reduce feature repeatability and geometric inliers [50,51]. Robust visual SLAM studies therefore assess localization residuals and trajectory continuity rather than image appearance alone [49,52,53].

**Figure 7:**
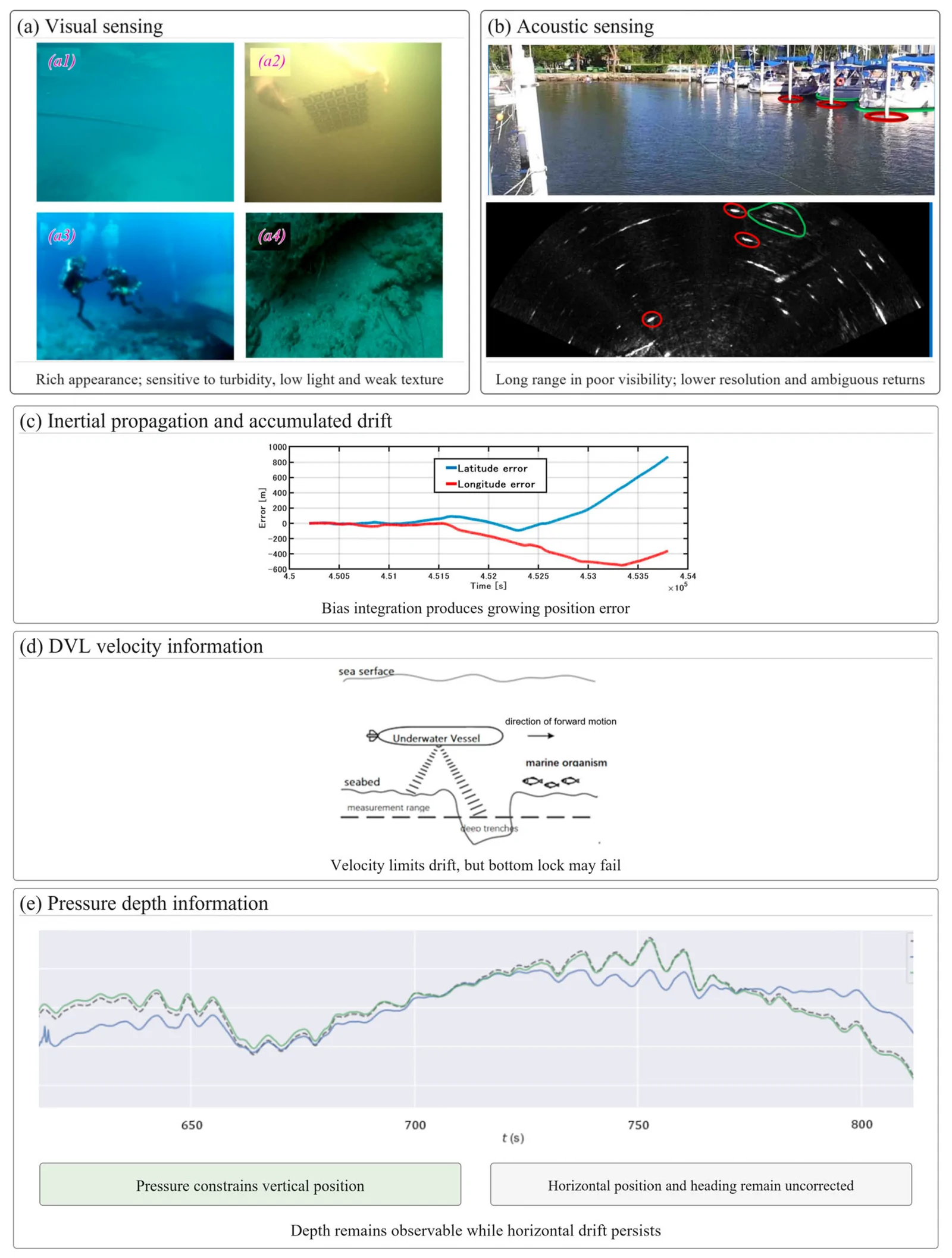
Panel (**a**) contains four visual-sensing examples, (**a1**–**a4**), showing different visibility and scene conditions; panel (**b**) shows an acoustic sensing scene and sonar image; panel (**c**) shows accumulated inertial drift, where the blue and red lines denote latitude and longitude errors, respectively; panel (**d**) illustrates DVL velocity measurement geometry; and panel (**e**) shows pressure-based depth information. Other colored or styled curves retain the meanings specified by their source legends. Source: Panels (**a**–**e**) were adapted from Ref. [59], Figure 3; Ref. [61], Figure 1; Ref. [52], Figure 11; Ref. [53], Figure 3; and Ref. [20], Figure 10, respectively. All source articles are licensed under CC BY 4.0. The panels were cropped, relabeled, and rearranged by the authors.

Acoustic sensing offers longer effective range and better visibility tolerance, but generally lower angular resolution, higher speckle, stronger multipath sensitivity, and more ambiguous association than optical sensing. Imaging sonar is therefore valuable for obstacle contours and structural boundaries in turbid water. Ultra-short baseline (USBL) or long baseline (LBL) systems can provide intermittent absolute position. Vision and sonar are complementary only when their measurements are synchronized and expressed in a common geometric model. Vision resolves short-range detail. Sonar preserves range and contour information. Inertial propagation bridges their unequal update rates [54,55]. Figure 7 summarizes this complementarity and the failure mechanisms that can reduce localization credibility [20,48,49,55,56].

The comparison indicates that no modality dominates all six dimensions. A low-cost, short-range system can use visual–inertial depth sensing when visibility and texture are verified; persistent turbidity or safety-critical operation requires acoustic or external absolute constraints; and DVL should be added when mission duration makes velocity drift consequential. The principal limitation is common-mode degradation, because multiple optical sensors may fail together in turbid water and multiple acoustic observations may be corrupted by the same shallow-water multipath. The engineering recommendation is therefore to configure heterogeneous redundancy around expected failure modes and to expose sensor validity and confidence to the estimator, planner, and safety manager [57].

### 3.2. Visual and Acoustic Sensing Front Ends and Degradation Handling

The technical question addressed in this section is how visual and acoustic front ends should convert degraded raw measurements into geometric constraints without creating confident but erroneous observations. Comparison should therefore cover geometric accuracy, robustness, processing cost, adaptation to turbidity and clutter, failure signatures, and onboard deployment requirements. Visual enhancement is useful only when it improves feature repeatability, inlier geometry, and pose continuity; methods such as ULL-SLAM and enhancement-assisted visual SLAM illustrate this task-oriented criterion [46,58]. Restoration that merely improves subjective appearance may amplify particles, bubbles, or backscatter and can increase false correspondences, so image-quality metrics must be evaluated together with trajectory error and estimator residuals [47,48,49,52,53,59,60,61].

The acoustic front end follows a different accuracy–cost tradeoff. Denoising, edge or target extraction, echo association, and scan matching can preserve structural constraints in low visibility, but acoustic measurements are strongly dependent on range, incidence angle, surface material, and scan organization. Motion priors can reduce the association search space and improve real-time feasibility, whereas dense matching or graph-based registration increases information use at greater computational cost. Sonar-inertial and visual-acoustic systems report association ambiguity and calibration sensitivity [62,63,64]. Side-scan and multibeam systems are additionally constrained by scan overlap and geometric degeneracy [54,56,65]. Mechanical profiling sonar remains vulnerable to insufficient elevation information and repetitive structure [55].

Dynamic fish, suspended matter, vegetation, and bubbles create a causal failure chain that is common to both front ends. Transient objects generate repeatable-looking features or echoes. These observations enter the static map, and the resulting false constraints bias localization. Temporal consistency tests, robust losses, cross-modal validation, and motion segmentation can reduce this risk, but they add latency and may also reject valid observations during aggressive motion. The applicable boundary is therefore determined by the relative time scales of vehicle motion, environmental dynamics, and sensor updates. For embedded deployment, quality indicators should be computed before expensive back-end optimization so that unreliable measurements can be rejected early [66,67,68].

Calibration and synchronization determine whether nominal sensor accuracy is retained after fusion. Fixed extrinsic errors, mounting flexure, and time offsets produce direction-dependent residuals that can resemble vehicle dynamics or environmental change; tighter coupling can then spread this bias across the complete state. Joint calibration in visual-acoustic and acoustic–visual–inertial systems improves observability when excitation and measurement diversity are sufficient, but it increases state dimension and computational burden [62,69]. Compact inland platforms should therefore combine predeployment calibration with online residual monitoring and should treat persistent bias as a health-state fault rather than ordinary measurement noise.

The synthesis is that visual processing should be prioritized for clear, textured, close-range inspection, acoustic processing for turbid or geometrically structured scenes, and cross-modal validation for environments containing dynamic organisms or repeated boundaries. Representative terrestrial studies show that lightweight detection and segmentation can support real-time classification, boundary extraction, and dynamic-object rejection [70,71,72]. However, these models require retraining and geometric validation under underwater scattering and color shift. Figure 7 should consequently be read as a configuration map: each modality supplies a different constraint, each has a recognizable degradation signature, and the estimator should accept only measurements whose quality, timing, and geometry satisfy the mission-specific threshold [73,74,75].

### 3.3. Fusion Levels, Estimation Frameworks, and Representative Methods

The technical question addressed in this section is which fusion architecture best converts heterogeneous observations into an accurate, robust, and computationally feasible state estimate. Loose coupling preserves modularity, predictable interfaces, and fault isolation, but discards correlations within raw measurements. Tight coupling retains feature, range, velocity, pressure, and inertial information and can remain observable when one subsystem cannot independently form a pose; however, it is more sensitive to calibration, synchronization, association errors, and computing load. Acoustic-VINS, AQUA-SLAM, and VIA-SLAM directly illustrate the information-versus-complexity tradeoff in tightly coupled acoustic, visual, inertial, pressure, and DVL fusion [69,76,77]. Sonar-assisted and hybrid visual–inertial systems show the same tradeoff under alternative sensor combinations [78,79,80,81].

The available engineering reports also illustrate the importance of separating update rate from end-to-end computation. Li et al. specified 200 Hz IMU, 0.5 Hz USBL, and 1 Hz DVL outputs in simulation and field-oriented evaluation. They ran the algorithms on an AMD Ryzen 7 5800U laptop with 16 GB RAM. Execution-time comparisons were presented, but no embedded CPU or GPU benchmark and no power measurement were reported [82]. In the AQUALOC-based visual–inertial pressure setting, the camera frequency was 20 Hz and the pressure measurement frequency was 5 Hz. Some frames lacked a paired pressure measurement [16]. These cases support reporting sensor update rates and hardware separately from estimator latency, memory, and power, because the latter cannot be inferred from the former.

Recursive filtering provides deterministic high-rate prediction, explicit covariance, and relatively low memory and latency, making it suitable for embedded platforms with stable state dimensions. Its main limitations are linearization sensitivity, dependence on noise assumptions, and limited retrospective correction. Sliding-window or graph-based optimization improves the use of asynchronous measurements, robust costs, calibration states, and delayed constraints, but solving time and memory grow with window size, factor count, and relinearization. Robust filtering and adaptive model sets address non-Gaussian disturbances and changes in DVL availability [83,84,85]. Outlier-aware graph optimization and mounting-error compensation target anomalous factors and systematic misalignment [82,86,87,88]. Event-triggered estimation addresses correlated noise and intermittent updates [89]. Together, these studies indicate that robustness depends more on representing loss of lock, outliers, correlated noise, and model transitions than on the estimator label.

A causal distinction is important. When sensing degradation merely increases random noise, covariance adaptation or robust weighting may be sufficient; when it removes an observable direction, optimization cannot recover the missing information without a new constraint. Loop closures and pressure or sonar factors can limit long-term drift, but erroneous associations may propagate globally, while aggressive marginalization can remove information needed for later correction [9,16,90]. Thus, higher nominal accuracy from a tightly coupled graph is meaningful only if worst-case latency, calibration stability, and outlier containment remain within the deployment envelope.

Learning-based components are most defensible when they estimate measurement quality, recover features, propose constraints, or select among model-based estimators. SM/VIO and switching visual–inertial frameworks demonstrate continuity under changing visual reliability, while independent diagnostic models can provide fault evidence outside the pose estimator [91,92,93,94]. Their environmental adaptability may exceed fixed thresholds, but domain shift and miscalibrated confidence are critical failure modes. For safety-related inland inspection, learned outputs should therefore be gated by geometric residuals and should not be the sole authority for accepting a state update [95,96,97].

A layered design uses filtering for high-rate propagation, a bounded window for multimodal correction, and global optimization only when retrospective consistency is required. Modular fusion favors fault isolation; tight coupling favors observability from partial measurements. Cross-domain systems support these interface principles [98,99,100], but underwater noise and failure modes require independent validation [101,102,103].

### 3.4. Typical Sensor Combinations and Scenario Adaptability

The technical question addressed in this section is which sensor combination provides sufficient localization credibility for a specified inland-water scene without unnecessary cost and integration burden. The IMU-depth-DVL combination offers accurate high-rate propagation and strong velocity-drift suppression when DVL lock is maintained; adding USBL supplies intermittent absolute position and improves long-duration boundedness. Its robustness is limited by bottom range, soft beds, beam occlusion, and acoustic anomalies, while its deployment cost and volume are higher than vision-based configurations [82,104]. It is therefore best suited to medium- or long-range missions in which sustained accuracy and predictable recovery justify the hardware and calibration effort.

Visual–inertial depth configurations provide the strongest cost-to-information ratio for clear-water, close-range, and textured inspection. Relative-depth assistance, pressure constraints, hybrid residuals, and loop closure improve scale and vertical stability [16,90,105]. Their characteristic failure is rapid loss of geometric constraints under turbidity, low illumination, repeated walls, or dynamic fish, which first lowers feature quality and then increases localization covariance and map inconsistency. Deployment should therefore include active illumination control, front-end quality scoring, and an inertial-depth fallback; acoustic support becomes necessary when visual degradation persists beyond the allowable drift interval.

Sonar-inertial configurations trade fine spatial accuracy for environmental adaptability in low visibility. They can exploit walls, dam faces, pipes, terrain, or artificial landmarks, but robustness depends on scan overlap, echo uniqueness, and multipath rejection. Parallel walls, repeated grids, flat beds, and shallow-water reflections can make one or more motion directions weakly observable, producing stable but incorrect matches [64,106,107]. Their computing cost ranges from moderate scan matching to heavier graph optimization. For deployment, sonar quality, geometric condition, and inertial drift should be monitored jointly, and a second constraint such as depth, DVL, or intermittent absolute position should be retained.

**Figure 8:**
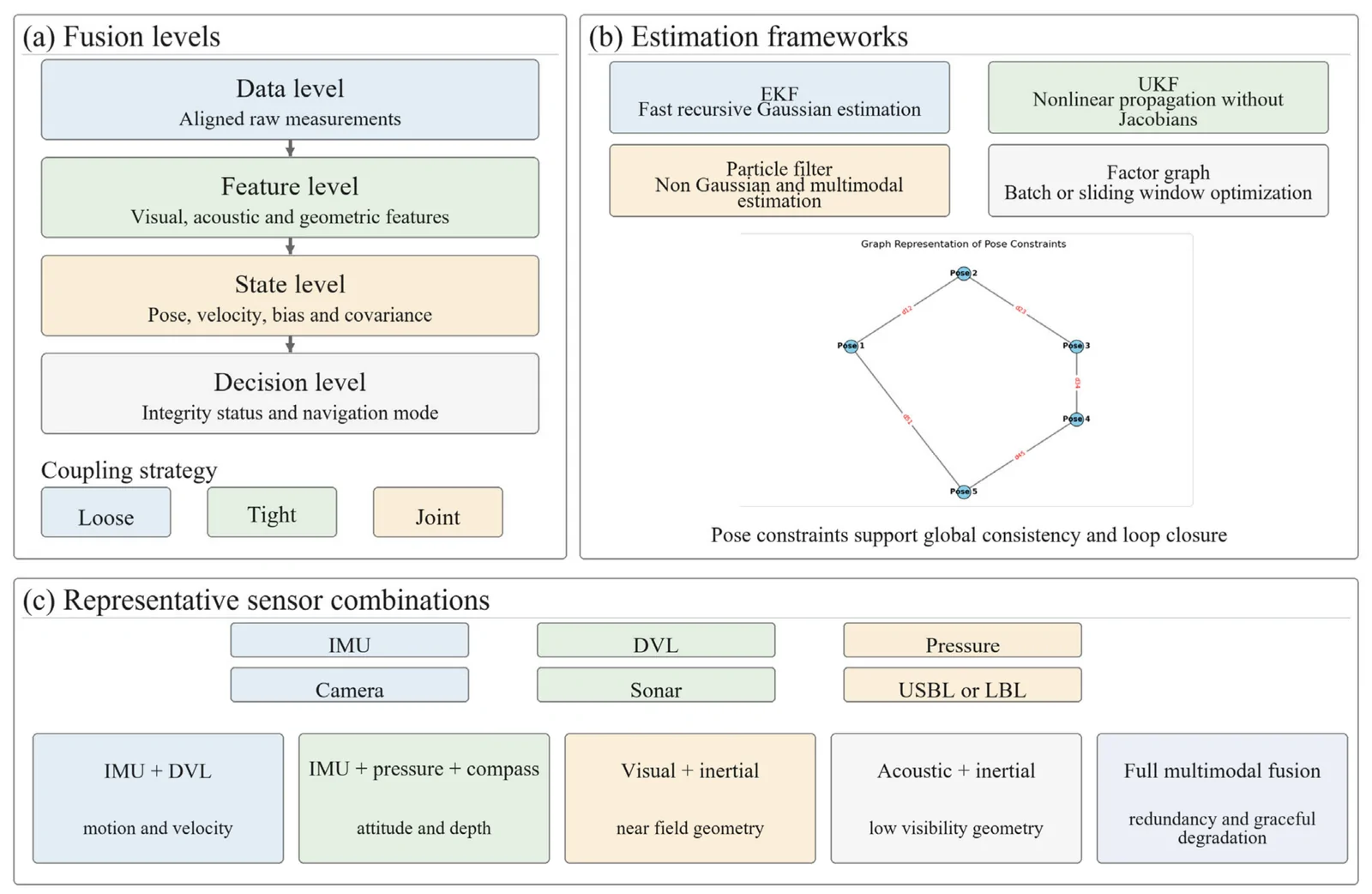
Multisensor fusion levels, estimation frameworks, and representative sensor combinations. Source: Summarized and redrawn by the authors based on Ref. [109] and Figure 2 in Ref. [110]; no source artwork is reproduced.

Full visual-sonar-inertial or visual-sonar-inertial-DVL combinations provide the greatest heterogeneous redundancy and can support localization, mapping, and obstacle perception across clear and turbid regions [69,76,77,108]. This benefit is conditional: unequal data rates, calibration, mounting stability, bandwidth, and optimization load create additional failure paths, and tight coupling can spread a biased modality into the global state. The configuration is therefore appropriate for high-consequence or highly variable missions, but not automatically for low-cost short sorties. Figure 8 organizes these choices by fusion level, estimation framework, and representative sensor set [109,110].

The comparative conclusion is to select sensors by the duration and consequence of their expected failure, not by modality count. For clear fish ponds and recoverable short missions, visual–inertial depth sensing is usually adequate. For persistent turbidity and near-wall work, sonar-inertial-depth sensing should be primary. For long-range mapping, DVL and intermittent absolute constraints become important. For critical facilities, heterogeneous optical, acoustic, velocity, and absolute observations should be combined with integrity monitoring. Semantic or RGB-depth processing may add useful landmarks in clear water, but should remain auxiliary after quality verification in degraded scenes [111,112,113]. The engineering implication is that every configuration needs a defined normal mode, degraded mode, and termination condition [114,115,116].

### 3.5. Robust Fusion and Localization Credibility Under Nonideal Conditions

The technical question addressed in this section is how a fusion system can remain credible when measurements are asynchronous, intermittent, biased, or completely unavailable. Average accuracy is insufficient for this purpose; robustness, computational latency, environmental adaptability, recognizable failure modes, and deployable recovery actions must be evaluated together. Timestamp alignment, interpolation, preintegration, and bounded reestimation can prevent temporal offsets from being interpreted as motion or calibration error, but they increase buffering and computation. The time-synchronization state should therefore be monitored continuously as part of navigation health, especially in tightly coupled systems [69,117].

Robust estimators must distinguish four conditions: isolated outliers, persistent bias, intermittent loss, and complete failure. Robust costs and innovation gating are effective for sparse anomalies; modular isolation limits cross-sensor contamination; and estimator switching maintains continuity when a modality becomes unavailable [82,91,92,117]. None of these mechanisms restores missing observability by itself. During DVL loss, for example, the system must explicitly increase uncertainty and enter a predictable degraded mode rather than retain the normal noise model [104]. The associated response should progress from adaptive weighting to mode switching and, when safety limits are reached, to deceleration, relocalization, surfacing, or return.

Environmental degradation affects localization credibility through a direct causal chain. Weak texture or backscatter reduces valid visual constraints; multipath or repeated geometry biases sonar association; loss of DVL lock removes velocity correction; and these effects enlarge or distort the estimated pose distribution. Tightly coupled estimation can exploit residual information, but it can also distribute a biased factor across all states [69]. Credibility assessment should therefore combine feature distribution, valid acoustic matches, DVL beam status, innovation statistics, information-matrix conditioning, computation delay, and cross-sensor consistency rather than relying solely on reported covariance.

**Figure 9:**
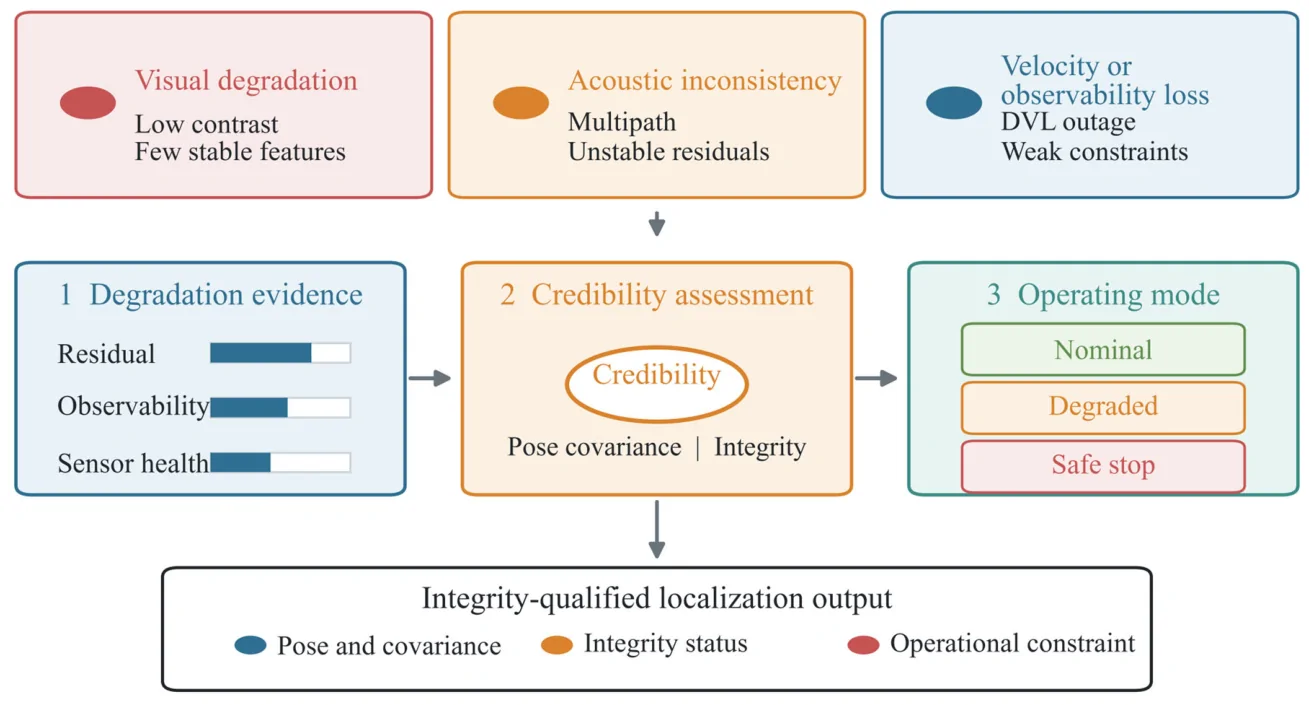
Robust fusion, confidence assessment, and degraded localization under adverse conditions. Source: Redrawn and synthesized by the authors from the robust-fusion and confidence-assessment concepts discussed in Refs. [119,120,121]; no source artwork is reproduced.

The synthesis is that a deployable localization output must include pose, covariance or protection information, active-sensor status, degradation level, and alarm state. Systems with reliable quality indicators and explicit degraded modes may be safer than nominally more accurate systems that fail silently. The current evidence base reports trajectory error more often than alarm limits, detection time, and integrity risk [109,118], so these remain validation limitations. Figure 9 summarizes the recommended chain from fault-tolerant fusion to confidence assessment and degraded localization [119,120,121]. For inland deployment, the planner should enlarge margins or seek informative regions as credibility decreases, and the mission manager should terminate operation when the required protection level can no longer be guaranteed.

### 3.6. Comparative Assessment of Fusion Localization and Sensor Configuration Schemes

Table 4 compares fusion families by accuracy, robustness, computation, environmental adaptability, failure mode, and deployment requirements. Filtering provides predictable latency; bounded optimization improves multisource use; robust estimation rejects abnormal DVL and acoustic updates [85,87,119]; switching isolates degraded modalities [91,92,104]; and model-data hybrids assist degradation recognition [93,94,122]. Reported metrics remain meaningful only with their reference, interval, and confidence assumptions.

A basic visual–inertial depth configuration suits short, clear missions; sonar-inertial-depth-DVL supports turbid or longer missions; safety-critical systems add heterogeneous redundancy, absolute updates, integrity monitoring, switching, and degraded modes. Capability rises with calibration, power, cost, software, and coupled failure paths. Detailed configurations remain in Table S1.

The principal causal criterion is coverage of failure modes. If visibility is good and mission duration is short, expensive acoustic positioning may offer little marginal benefit; if turbidity is persistent or boundaries are repetitive, acoustic range, DVL velocity, or absolute updates become more valuable than deeper visual fusion. Dataset replay using Tank, AQUALOC, natural-scene AUV data, and pipeline simulations can reveal algorithmic degradation, but it does not reproduce every shallow-water reflection, biological disturbance, or recovery constraint [50,51,122,123]. Configuration acceptance must therefore progress from reproducible data tests to pool and representative inland-water trials.

Computational feasibility is also conditional. Filtering is typically most deterministic, whereas graph optimization, visual front ends, and learned components add solving, image, memory, or GPU load. Reported platforms include a Ryzen 7 5800U with 16 GB RAM [82] and an i9-9900K with 16 GB RAM and RTX 2080 Ti [52], without comparable end-to-end timing. Jetson feasibility therefore requires target-vehicle latency, jitter, memory, power, and degraded-mode tests; source details are retained in Table S2.

Overall, the preferred scheme is the least complex configuration that preserves heterogeneous constraints through the mission’s credible operating envelope. Low-risk systems should prioritize deterministic filtering and recoverability; variable or turbid environments justify bounded optimization and acoustic support; and critical infrastructure requires integrity outputs, independent fault detection, and a verified fallback. Figure 9 makes this selection principle explicit: fusion quality must be converted into confidence, confidence must control estimator mode and planning margins, and unresolved loss of credibility must trigger a safe mission response.

## 4. Structural Design, Functional Payloads, and Platform Integration of Underwater Robots

Reliable estimates become repeatable mission capability only when implemented on a suitable vehicle, propulsion system, payload, power system, and computer. This chapter examines how those hardware choices constrain sensing quality, feasible motion, and fault handling.

### 4.1. Platform Types and Mission Adaptation of Underwater Robots

The technical question addressed in this section is which platform type can deliver the required sensing, localization, planning, and control performance within the mission’s range, recovery, and failure constraints. Streamlined AUVs support efficient long-range motion but provide weaker hovering and confined-space maneuverability [3,17,18,19]. Open-frame ROVs provide payload flexibility, station keeping, operator supervision, and high-bandwidth tethered communication [23,124]. Their limitations include tether drag, entanglement risk, and restricted reach. Cooperative USV-underwater systems add surface positioning and relay capacity, but increase synchronization, communication, and task-allocation complexity [44]. Platform accuracy and robustness therefore arise from the complete vehicle-support system rather than autonomy level alone.

Environmental adaptability separates these platform classes more clearly than nominal speed. Fish ponds, lock chambers, and culverts favor compact vehicles with low-speed control, lateral motion, and easy recovery [20,44]. River transects and reservoir surveys favor efficient AUVs with sufficient propulsion reserve [20,44]. Cooperative systems are useful when surface GNSS updates or data relays materially reduce localization and communication risk [20,44]. Failure modes also differ: tethered systems risk entanglement and drag-induced tracking error, untethered systems risk communication loss and difficult recovery, and cooperative systems add intervehicle dependence.

Larger platforms usually offer more payload and computation but increase collision consequence, launch burden, and life-cycle cost. Offshore near-wall evidence [1,22,23,42] requires revalidation for shallower water, tighter boundaries, stronger multipath, and human interaction. Motion envelope, sensing field of view, stopping distance, and recovery should be fixed before algorithm complexity.

Compact ROV or hybrid platforms suit confined recoverable inspection, streamlined AUVs suit long transects, and surface-underwater cooperation is justified when positioning and communication gains exceed coordination cost. Cross-domain design studies support task-based hardware and autonomy matching [111,125,126], but underwater configuration must follow current, payload, recovery, and failure consequence.

### 4.2. Hull Structure, Propulsion Layout, and Motion Capability

The technical question addressed in this section is how hull form and thruster layout determine the accuracy, robustness, energy cost, and feasible trajectories of the navigation system. Streamlined hulls reduce drag and support efficient forward survey, but their low-speed turning and station-keeping capability may be limited. Open-frame structures accept modular payloads and multiple lateral or vertical thrusters, improving hovering and near-wall control, but increase drag, flow disturbance, and exposed-component risk. Modular designs can balance these properties, although every reconfiguration changes mass distribution, hydrodynamics, and calibration [112].

Propulsion layout must be compared through controllable degrees of freedom, thrust authority, saturation, low-speed efficiency, redundancy, and power demand. A dual-horizontal plus vertical arrangement is economical for transects and depth regulation; vectored multithruster layouts improve lateral motion and attitude decoupling for walls, dams, and bridge piers but increase allocation complexity and energy consumption. Dead zones, mounting errors, shallow-water effects, near-wall flow, and tether drag cause planned kinematic paths to become dynamically infeasible. Turning radius, acceleration, braking distance, attitude rate, and residual thrust after failure must therefore enter the planner and controller as measured constraints [113,124,127,128].

**Figure 10:**
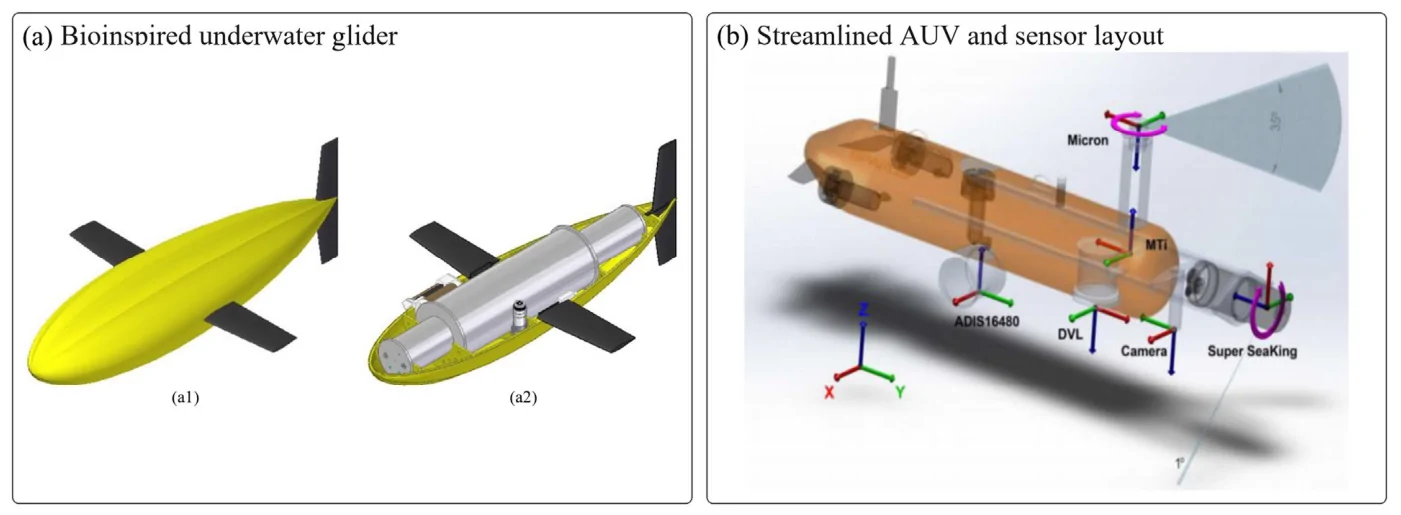

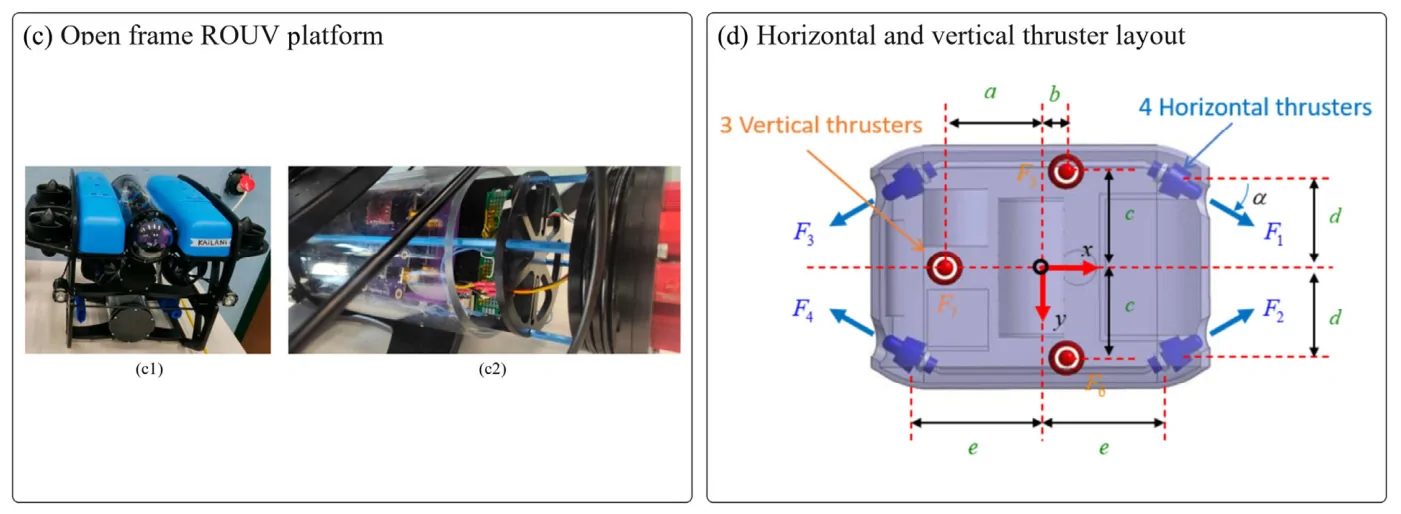
Panel (**a**) contains (**a1**), the external form of a bioinspired underwater glider, and (**a2**), its internal structure; panel (**b**) shows a streamlined AUV and sensor layout; panel (**c**) contains (**c1**), the external open-frame ROV platform, and (**c2**), its internal structure; and panel (**d**) shows the horizontal and vertical thruster layout. In panel (**d**), a–e denote geometric spacing parameters, alpha denotes the thruster installation angle, F1-F6 denote thrust vectors, and x and y denote the body-frame axes. Source: Panels (**a**–**d**) were adapted from Ref. [126], Figure 11; Ref. [127], Figure 3; Ref. [128], Figure 2; and Ref. [133], Figure 2, respectively. All source articles are licensed under CC BY 4.0. The panels were cropped and rearranged by the authors.

The synthesis is that hull and propulsion should be selected by mission coverage per unit energy, trackability near obstacles, payload visibility, and recoverability rather than drag alone. Lightweight low-disturbance platforms suit fish ponds and routine monitoring, whereas critical-facility inspection justifies greater mass, multiaxis thrust, and propulsion redundancy. The main limitation of compact platforms is restricted energy and computation; the main limitation of highly actuated platforms is integration and power burden. Figure 10 summarizes the corresponding platform, hull, and propulsion choices. Engineering acceptance should verify the same motion limits used by planning and control under current, near-wall, and single-thruster-degradation conditions.

### 4.3. Navigation Sensing Systems and Mission Functional Payloads

The technical question addressed in this section is how navigation sensors and mission payloads should be arranged so that measurement accuracy and data-product quality are preserved on the actual vehicle. Alternative arrangements should be compared through accuracy, robustness, computational and power cost, environmental adaptability, mounting-related failure modes, and deployment and maintenance conditions. The IMU should remain close to the rotation center, cameras require a rigid baseline and controllable illumination, sonar requires an unobstructed acoustic field, and DVL requires valid incidence and range. Visual-acoustic and visual–inertial systems show that extrinsic and temporal errors generate systematic residuals [16,62,69]. Inertial navigation system (INS)/DVL fusion studies likewise show that mounting misalignment must be modeled explicitly [82,86]. Tight coupling can propagate these biases through the state estimate [76,77,90].

Mission payloads impose additional navigation conditions. Water quality probes require speed and dwell times compatible with sensor response; cameras and lights determine wall distance and viewing angle; manipulators require higher station-keeping accuracy and thrust reserve; and aquaculture observation must limit illumination, acoustic, and wake disturbance. Compared across deployment conditions, imaging payloads demand geometric stability and visibility, sampling payloads demand temporal registration, and intervention payloads demand accurate force and pose control. Recent inspection systems increasingly produce pose-referenced three-dimensional models [129,130]. Pipeline and dam studies further associate defect classes and quantitative dimensions with vehicle pose [131,132,133].

The causal link is that payload requirements shape the path, the path shapes vehicle attitude and speed, and these states in turn change sensing quality. A camera trajectory that violates standoff or incidence constraints may reduce defect-detection accuracy even when vehicle position error is small; conversely, a slow sampling dwell can increase energy consumption and current-induced control effort. Raw measurements, fused pose, covariance, health flags, and trigger events should therefore share a common timestamp and coordinate system. Without this traceability, localization uncertainty cannot be propagated into the confidence of the final water quality or defect map.

Payload and navigation sensing should be treated as one calibrated measurement system. Edge processing reduces communication load but increases heat, power, and scheduling pressure. Cross-domain systems support unified triggering and sensing-control interfaces [134,135,136,137], whose underwater use still requires validation against sealing, scattering, acoustic interference, and link limits [138,139,140].

### 4.4. Power Supply, Communication, and Embedded Computing Systems

The technical question addressed in this section is how power, communication, and embedded computation constrain navigation accuracy, robustness, and mission continuity. Propulsion, lighting, sonar, and computation compete for a finite energy budget, and minimum distance does not necessarily mean minimum energy because hovering, repeated acceleration, and current rejection can dominate consumption [112,124,127]. The planner should reserve energy for return, surfacing, and fault handling and should update the feasible mission region using battery state, instantaneous load, current, and remaining observation value.

Communication options occupy different deployment envelopes. Acoustic links provide range but have low bandwidth and are affected by latency and shallow-water multipath. Optical links provide bandwidth but require short range, line of sight, and clear water. Tethers support real-time video and takeover but introduce drag and entanglement. Surface relays extend external communication but add cooperative-system dependencies [44]. Because every link can become unavailable, attitude, depth, obstacle avoidance, return, and safe termination must remain onboard. Communication should update mission intent, not serve as a continuous prerequisite for the control loop.

**Figure 11:**
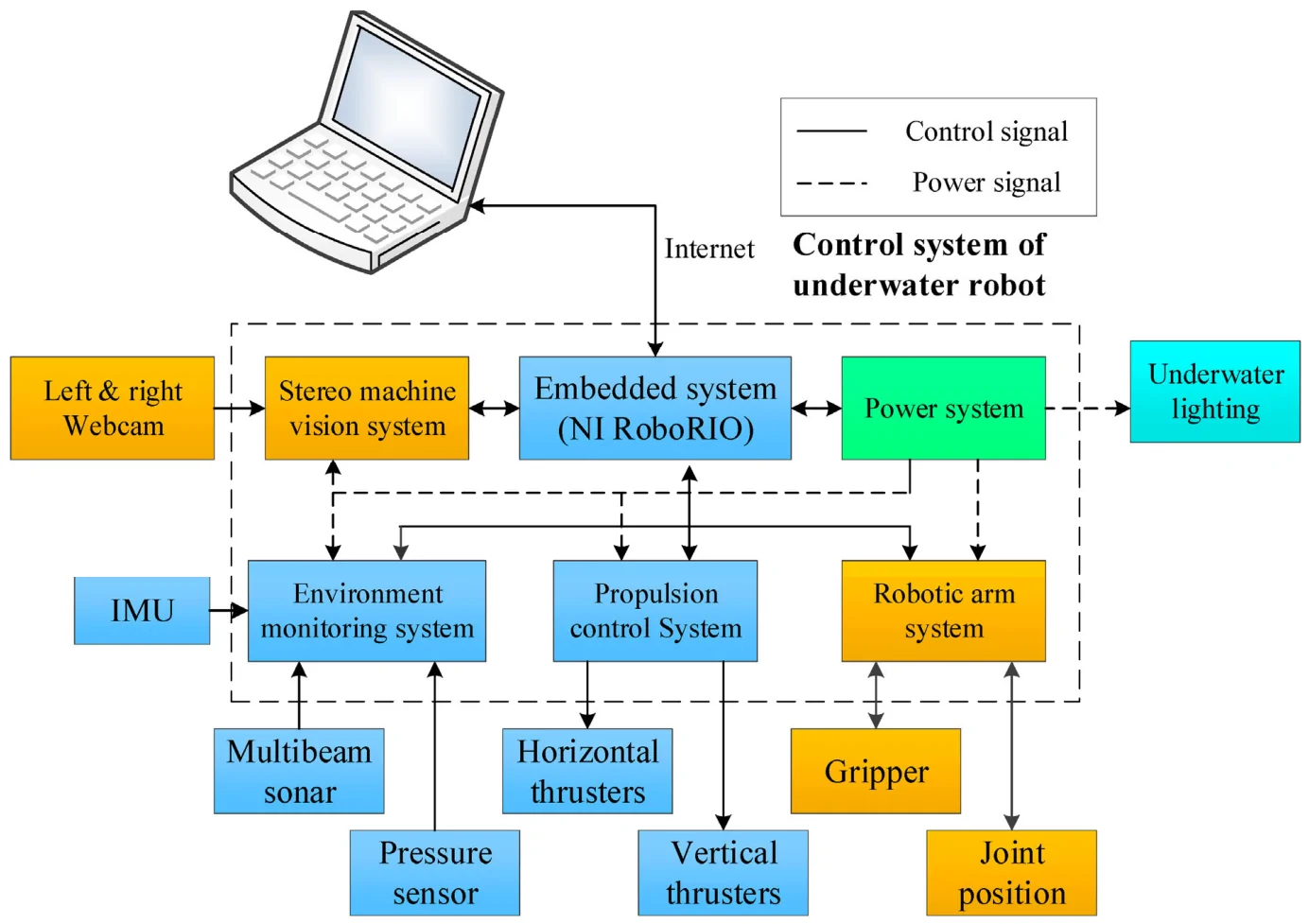
Navigation sensing, mission payload, power, communication, and embedded computing subsystems. Source: Adapted from Ref. [141], Figure 2, under CC BY 4.0. The layout and labels were reorganized by the authors.

Embedded computation should be compared by worst-case latency, jitter, peak memory, power, thermal behavior, and fault isolation rather than average frame rate. Control requires deterministic timing, fusion and sonar require sustained throughput, and learned perception may require GPU resources. Computational overload first delays perception and localization, then makes the map stale, reduces replanning time margin, and finally increases tracking and collision risk. Task priorities should therefore preserve attitude, depth, basic obstacle avoidance, return, and logging before high-cost mapping or semantic processing. Figure 11 shows the hardware relationships among sensing, payload, power, communication, computation, and actuation [141].

The synthesis is that energy, communication, and computation are active navigation constraints rather than supporting utilities. Low-cost short missions can favor reduced-rate perception and deterministic filtering; long-range missions require energy-aware planning and communication-tolerant autonomy; and safety-critical missions require resource monitoring, task shedding, and independent fallback control. Experience from autonomous agricultural machinery similarly shows that continuous operation depends on coordination among these subsystems [142]. The engineering recommendation is to test the minimum safe closed loop under low battery, communication interruption, thermal throttling, and computational overload before adding optional mission functions.

### 4.5. Modular Integration, Calibration Synchronization, and Reliability Design

The technical question addressed in this section is how modular integration can preserve calibration, timing, reliability, and fault isolation after sensors or payloads are replaced. Standard mechanical, electrical, communication, coordinate, timestamp, quality-flag, and health-state interfaces improve maintainability, but physical replacement changes mass, buoyancy, extrinsic parameters, power quality, and sometimes thruster allocation [112,117]. Modularity therefore reduces integration cost only when every module has an explicit acceptance test and a traceable calibration version.

Calibration methods differ in accuracy, computational cost, and observability requirements. Offline calibration is repeatable and inexpensive during operation but cannot capture mounting flexure or maintenance-induced changes; online estimation can compensate gradual drift but enlarges the state and may become weakly observable. Camera intrinsics, intersensor extrinsics, thrust curves, pressure zero, magnetic bias, and time offsets should be validated through laboratory calibration, prelaunch self-check, online residual monitoring, and postmaintenance recalibration [62,69,86]. Persistent residual bias should trigger inspection rather than indefinite covariance inflation.

Reliability is limited by sealing, corrosion, fouling, window contamination, sensor drift, thermal load, and thruster entanglement. These faults propagate differently: optical fouling reduces perception before localization, connector faults may remove an entire modality, and thrust loss converts a feasible trajectory into an untrackable one. Homogeneous redundancy may fail simultaneously under turbidity, bubbles, or electromagnetic interference, whereas heterogeneous redundancy is useful only if faults are detectable and sufficiently independent. Health thresholds must therefore be paired with continuation, degradation, surfacing, or return actions.

The comparative conclusion is that a modular platform should be evaluated by restoration time, calibration repeatability, fault containment, and postreplacement mission performance, not by connector standardization alone. Integrated sensing-control experience supports joint verification of mechanical, electrical, and software interfaces [143]. For deployment, each maintenance event should trigger a concise acceptance sequence covering watertightness, coordinate frames, clock alignment, sensor residuals, thrust response, and safe-state execution. This limits the possibility that a small hardware change silently reduces localization credibility or control authority.

### 4.6. Platform Configuration Schemes for Typical Inland Missions

Mission requirements should determine platform accuracy, robustness, cost, and failure handling. Fish pond monitoring favors light launch and recovery; rivers require current authority and obstacle tolerance; reservoir mapping requires endurance and drift control; and critical facilities require high integrity and auditable recovery.

Configuration should proceed from mission boundary to platform, propulsion, primary localization constraint, auxiliary sensing, payload, power and communication, and safety response. Persistent turbidity favors sonar; unstable DVL lock requires alternative constraints; severe communication consequences require onboard avoidance and autonomous return.

Added accuracy is useful only when computation, calibration, maintenance, and recovery remain feasible. Cross-domain integration studies offer interface and staged-test practices [144,145,146], but underwater acceptance must reproduce visibility, multipath, current, pressure, and recovery conditions.

Table 5 compares task-conditioned platform configurations and their corresponding sensing, mobility, and verification priorities.

**Figure 12:**
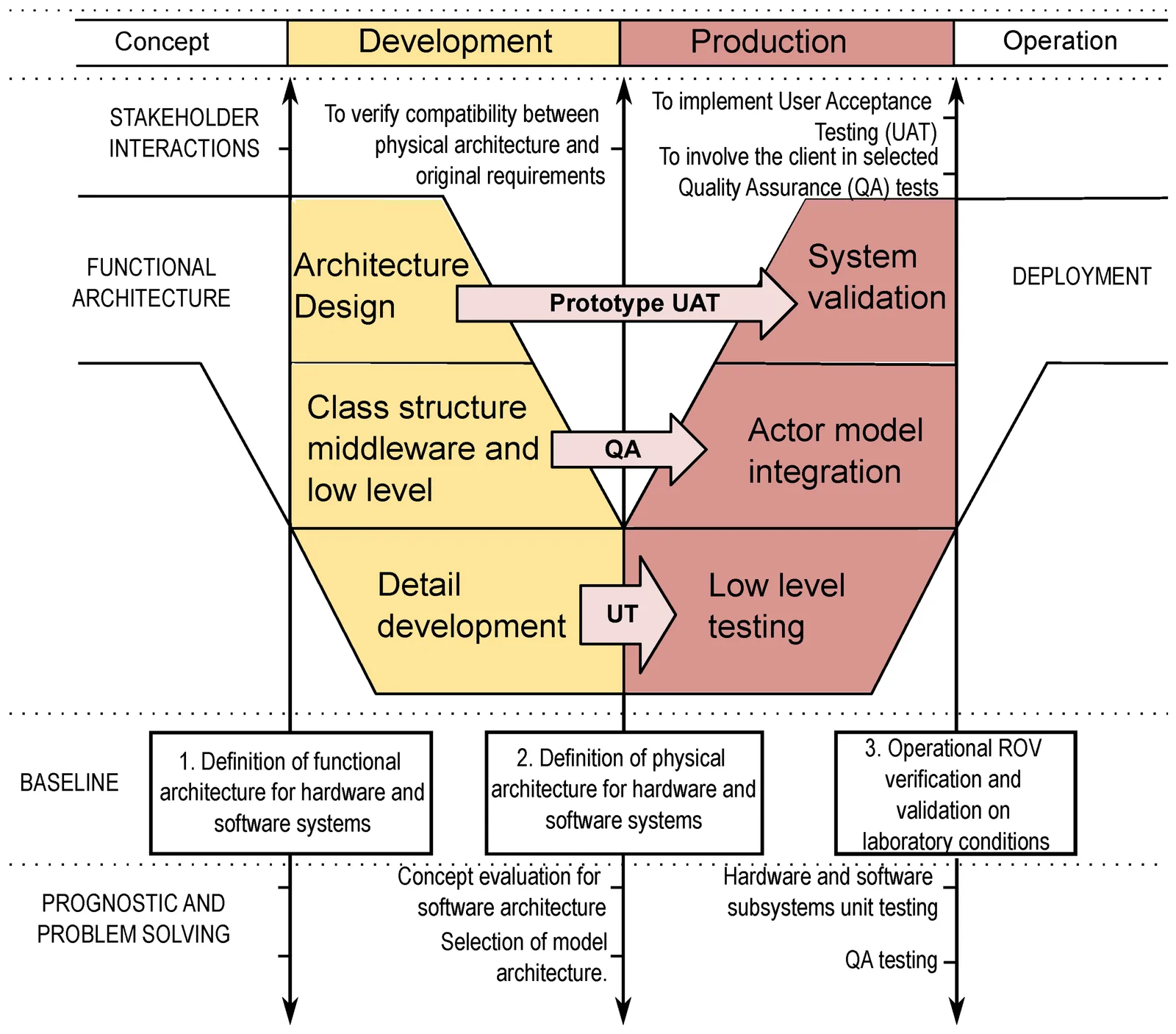
Requirement decomposition, system integration, and verification workflow for underwater robot platforms. Source: Reproduced from Ref. [148], Figure 3, under CC BY 4.0.

The preferred platform is the minimum configuration with a detectable and controllable degradation path: simple and recoverable for ponds, endurance and drift control for reservoirs, and integrity, redundancy, and fail-safe recovery for critical infrastructure. Figure 12 traces each subsystem choice to mission risk and validation.

## 5. Localization-Uncertainty-Aware Path Planning and Dynamic Obstacle Avoidance

Path planning must convert pose credibility, maps, platform limits, and mission objectives into executable trajectories. This chapter compares global and local planning, dynamic replanning, trajectory feasibility, and the propagation of localization uncertainty into risk.

### 5.1. Inspection Planning Problems, Map Representation, and Multiobjective Evaluation

The technical question addressed in this section is how an inland inspection mission should be represented so that planning accuracy, robustness, computational cost, environmental adaptability, failure modes, deployment conditions, and data-product requirements are evaluated within one problem formulation. Point-to-point navigation prioritizes safe arrival, coverage planning prioritizes omission and overlap, boundary following prioritizes standoff and viewing geometry, fixed-point sampling prioritizes residence and registration, and reinspection prioritizes repeat-arrival accuracy [40,41]. Because a mission may combine these modes, a unified task graph should encode route segments, sampling events, exclusion regions, information targets, recovery points, and the validity period of each constraint.

Map representation determines both planning resolution and failure behavior. Two-dimensional grids support efficient and interpretable search but discretize geometry. For shallow water with limited vertical freedom, 2.5-dimensional cost maps are well-suited. Voxels and point clouds represent confined three-dimensional structures at a higher memory and update cost. Topological graphs scale to long missions but provide less local detail. Semantic or spatiotemporal maps can distinguish walls, nets, vegetation, moving objects, and stale observations. Localization uncertainty directly affects all representations. Pose error broadens occupied regions, misregisters repeated observations, and can turn an apparently free corridor into an unsafe one.

Planning objectives should therefore be ordered rather than indiscriminately weighted. Collision probability, platform reachability, minimum clearance, and return energy should form hard or chance constraints; path length, duration, smoothness, flow cost, sensing benefit, communication opportunity, and mission priority can then be optimized within the feasible set [13,153,154,155]. The comparison indicates that no map or objective set is universally optimal: regular ponds favor compact grids and coverage costs, long reservoirs favor hierarchical or topological planning, and confined structures require three-dimensional geometry and sensor-view constraints. The limitation is that richer maps and predictive objectives increase computation and model dependence. The engineering recommendation is to select the least detailed representation that preserves the mission’s safety and observation constraints and to attach timestamps, source confidence, and localization uncertainty to map updates.

### 5.2. Graph Search, Incremental Search, and Sampling-Based Planning

The technical question addressed in this section is which deterministic or sampling planner can produce a sufficiently accurate and executable route within the available map and computational budget. Dijkstra provides a reproducible shortest-path baseline but expands broadly. A-star (A*) reduces expansion through an admissible heuristic. D-star Lite (D* Lite) reuses prior search when local map costs change [16,17,156,157,158]. These graph methods are interpretable and robust on structured maps, but accuracy and computational cost depend on grid resolution, obstacle inflation, and directional penalties. Coarse maps improve speed but may remove narrow feasible corridors, whereas fine maps increase memory and update latency.

Probabilistic roadmap (PRM), rapidly exploring random tree (RRT), RRT*, and related sampling methods avoid exhaustive grid traversal and adapt to continuous or higher-dimensional state spaces. RRT often finds an initial feasible route quickly, while RRT* improves path quality asymptotically. Both can suffer from random variability, narrow-passage inefficiency, and uncertain worst-case latency. Motion primitives, current-aware bias, dynamic feasibility checks, and risk screening improve deployment relevance but add model and computation requirements [159]. In regular fish ponds or channels, a two-dimensional or 2.5-dimensional graph search is generally more efficient. Sampling becomes more attractive for complex three-dimensional structures or nonholonomic motion constraints. In these established algorithm names, the asterisk is part of the conventional notation rather than a separate mathematical operator.

The causal boundary between these families lies in representation and update frequency. Graph search is accurate only to the discretized cost map and can replan predictably when changes are localized; sampling explores continuous free space but may not repeat the same solution under identical geometry. Localization error affects both by shifting collision checks and obstacle inflation, while stale perception affects incremental reuse and sampled feasibility. Fair comparison therefore requires the same map, start and goal, obstacle density, uncertainty, dynamic constraints, random seeds, and computing budget, with success rate, worst-case time, repeatability, path cost, and postprocessing reported.

**Figure 13:**
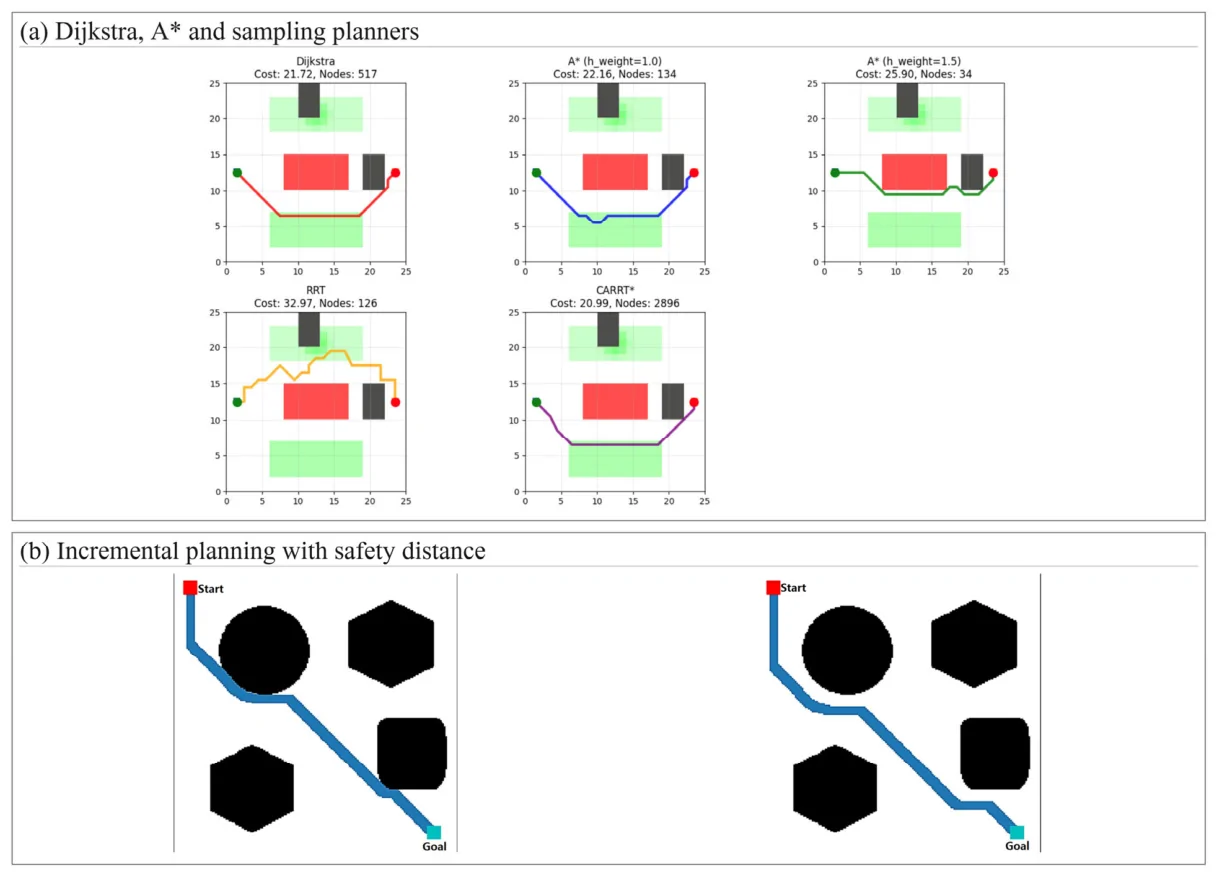
Representative path comparison of graph-search, incremental-search, and sampling-based planners. Source: Panel (**a**) adapted from Ref. [159], Figure 2, and panel (**b**) adapted from Ref. [163], Figure 8. Both cited source articles are licensed under CC BY 4.0, as verified from the publisher and license records archived with this revision. The panels were cropped and rearranged by the authors. In panel (**a**), colored curves represent planner-specific paths and the green and red markers indicate the start and goal; in panel (**b**), the blue line denotes the planned route, the red and cyan squares mark the start and goal, and the black shapes denote obstacles. These conventions are retained from the source figures.

The synthesis favors hierarchical use rather than a universal winner. Apply A* or incremental search to regular maps and changing local costs, use PRM or RRT variants when dimensionality and dynamics make grids inefficient, and combine a coarse global corridor with short-horizon feasible motion generation. Improved D* Lite and hybrid planning confirm the value of reusing prior search information [160,161]. Figure 13 compares representative path characteristics. For deployment, obstacle inflation must include localization uncertainty and stopping distance, and a deterministic fallback should be retained whenever sampling or postprocessing exceeds its time budget [162].

### 5.3. Intelligent Optimization, Learning-Based, and Hybrid Planning Methods

The technical question addressed in this section is where intelligent optimization and learning add value beyond deterministic search without weakening real-time and safety guarantees. Genetic, particle-swarm, ant-colony, differential-evolution, and related methods can optimize nonconvex combinations of length, energy, smoothness, and current, but accuracy and convergence depend on population size, parameter settings, random seeds, and termination criteria [164]. Their computational cost and worst-case delay make them most suitable for offline route design, low-rate replanning, or initialization of constrained local optimization rather than sole responsibility for safety-critical avoidance.

Reinforcement and imitation learning can represent dynamic interactions and unmodeled flow effects, but robustness is limited by simulation-to-reality shift, incomplete coverage of rare hazards, reward misspecification, and unreliable confidence [165,166,167,168]. Sensing degradation introduces an additional causal risk: a policy trained on accurate observations may respond aggressively to biased pose or obstacle estimates, while its internal confidence may not reveal the error. A safer deployment boundary is to use learning for flow prediction, obstacle-motion prediction, sensing-quality estimation, or heuristic guidance and to retain explicit collision, reachability, and return-energy constraints in the final decision layer.

Hybrid planning combines global search accuracy and interpretability, local optimization for dynamic feasibility, and learning for prediction or adaptation. A global corridor can constrain model predictive planning, while localization credibility adjusts obstacle margins and learning outputs propose rather than authorize actions. This arrangement increases software and computing complexity, but it provides clearer fault containment: if prediction degrades, the system can revert to deterministic costs and a conservative speed profile. The relevant comparison is therefore interface transparency, worst-case computation, fallback behavior, and closed-loop success, not the number of constituent algorithms.

Intelligent optimization is justified when it improves a bounded prediction or multiobjective subproblem. Cross-domain coverage and steering studies provide transferable structures [157,169,170], but underwater deployment must revalidate current, three-dimensional motion, communication, localization credibility, and return energy [171].

### 5.4. Local Obstacle Avoidance, Dynamic Replanning, and Trajectory Feasibilization

The technical question addressed in this section is how local planning should transform uncertain, delayed observations into a collision-free trajectory that the platform can execute in real time. Artificial potential fields have low cost but may oscillate or become trapped; dynamic-window methods explicitly include stopping and velocity limits but are most natural for low-dimensional motion; velocity-obstacle methods address moving targets but depend on motion prediction. Underwater deployment adds three-dimensional relative motion, current, sensor blind zones, localization delay, and irregular actuation [172,173,174]. Accuracy must therefore be judged by clearance and tracking feasibility, not only by geometric path length.

Dynamic replanning must distinguish transient detections, predictable moving objects, and persistent structural changes. Writing fish schools or floating matter into a permanent map creates false blockage; ignoring them creates collision risk. Short-term tracks and time-indexed occupancy provide a middle layer, while global replanning should be reserved for persistent corridor blockage, repeated local failure, or degraded localization. Hysteresis and minimum holding times improve robustness by preventing oscillatory replanning, but overly conservative thresholds can delay necessary response. Prediction and gradient-based optimization show how obstacle forecasts and collision costs jointly shape the local trajectory [175].

A directly reported replanning timing example is available from the UX-1 software-in-the-loop experiment. The vehicle speed was limited to 0.1 m/s, and an obstacle was introduced at a 1 m distance. Collision checking required approximately 1.3 s to detect the invalidated path. A new path request was sent 0.7 s later, and the new path was planned in approximately 2.3 s. The vehicle traveled 10.2 cm during the first 1.3 s of latency [37]. This is a simulated confined-tunnel case with a specific collision-checking and planning stack. It should therefore be treated as a traceable timing example rather than a general replanning benchmark. The underwater planning literature reviewed here does not provide a common real-water value for end-to-end replanning latency. Such values should remain reported as not available when the original study does not measure them.

**Figure 14:**
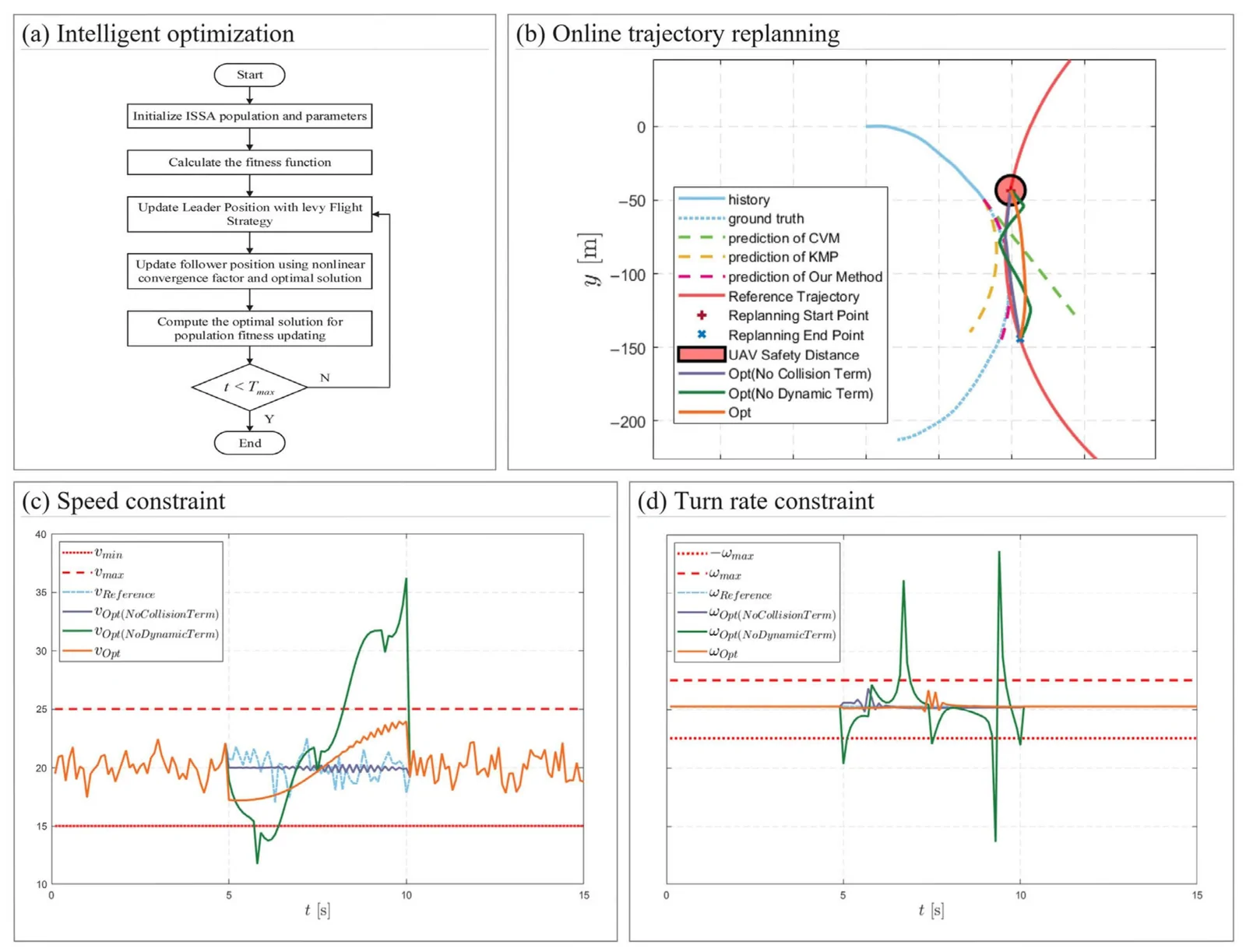
Intelligent optimization, online dynamic replanning, and trajectory-feasibility constraints. Source: Panel (**a**) adapted from Ref. [164], Figure 3; panels (**b**–**d**) adapted from Ref. [175], Figures 3, 5, and 6, respectively. Both cited source articles are licensed under CC BY 4.0, as verified from the publisher and license records archived with this revision. The panels were cropped and rearranged by the authors.

Trajectory feasibilization closes the causal gap between planning and control. Curvature, speed, acceleration, attitude rate, thrust saturation, braking distance, and sensor viewing geometry must be checked against the identified platform limits from Chapter 4. If this step is omitted, the controller may cut corners or saturate, reducing clearance even though the planned path is collision-free. Splines and motion primitives are computationally efficient but depend on chosen bounds; short-horizon optimization handles coupled constraints more directly but raises latency and solver-failure risk. Figure 14b–d illustrate the relationship among replanning, velocity, and steering constraints [175].

The comparison indicates that local safety is jointly determined by sensing update rate, localization credibility, motion prediction, computation time, and braking authority [176,177,178]. Potential fields suit simple recoverable scenes, dynamic-window or motion-primitive methods suit platforms with well-characterized dynamics, and probabilistic or optimization-based methods are preferable when moving obstacles and uncertainty dominate. Figure 14 summarizes the complete route from intelligent optimization to feasible trajectory generation. The engineering recommendation is to enlarge margins and reduce speed as uncertainty or latency increases. Global replanning should be triggered only under persistent conditions. An emergency stop, hover, or retreat action should remain independent of the nominal local planner [179,180].

### 5.5. Coordination Between Localization Uncertainty and Path Planning

The technical question addressed in this section is how localization credibility should alter mapping, path selection, speed, and safety actions. Candidate interfaces should be compared through accuracy, robustness, computational cost, environmental adaptability, uncertainty-related failure modes, and deployment conditions. Treating the estimated pose as exact shifts both the robot footprint and observed obstacle boundaries. Covariance inflation, protection levels, belief-space propagation, collision probability, and chance constraints provide progressively richer ways to represent this risk [181]. Their accuracy depends on covariance consistency and map-registration quality, while their computational cost increases from simple obstacle inflation to distribution-aware optimization. Planning metrics are comparable across studies only when hardware, map resolution, mission scale, and uncertainty assumptions are consistent.

Uncertainty propagates causally through the closed loop. Degraded sensing weakens or biases pose constraints. Localization uncertainty then broadens and misaligns map occupancy. The planner either underestimates clearance or becomes excessively conservative. The controller receives a trajectory whose risk or curvature may no longer match the true state. Active localization reverses part of this chain by selecting regions with texture, distinctive sonar geometry, external positioning, or informative excitation. Surfacing, boundary approach, small observability maneuvers, and return to known landmarks can trade distance for information gain [182,183]. These actions remain appropriate only when the maneuver itself is safe and energy-feasible.

A deployable coordinator should use explicit states: normal credibility permits efficient planning, reduced credibility enlarges margins and favors informative routes, and an alarm triggers relocalization, surfacing, return, or termination. Table 6 compares planning families within their capability boundaries.

**Figure 15:**
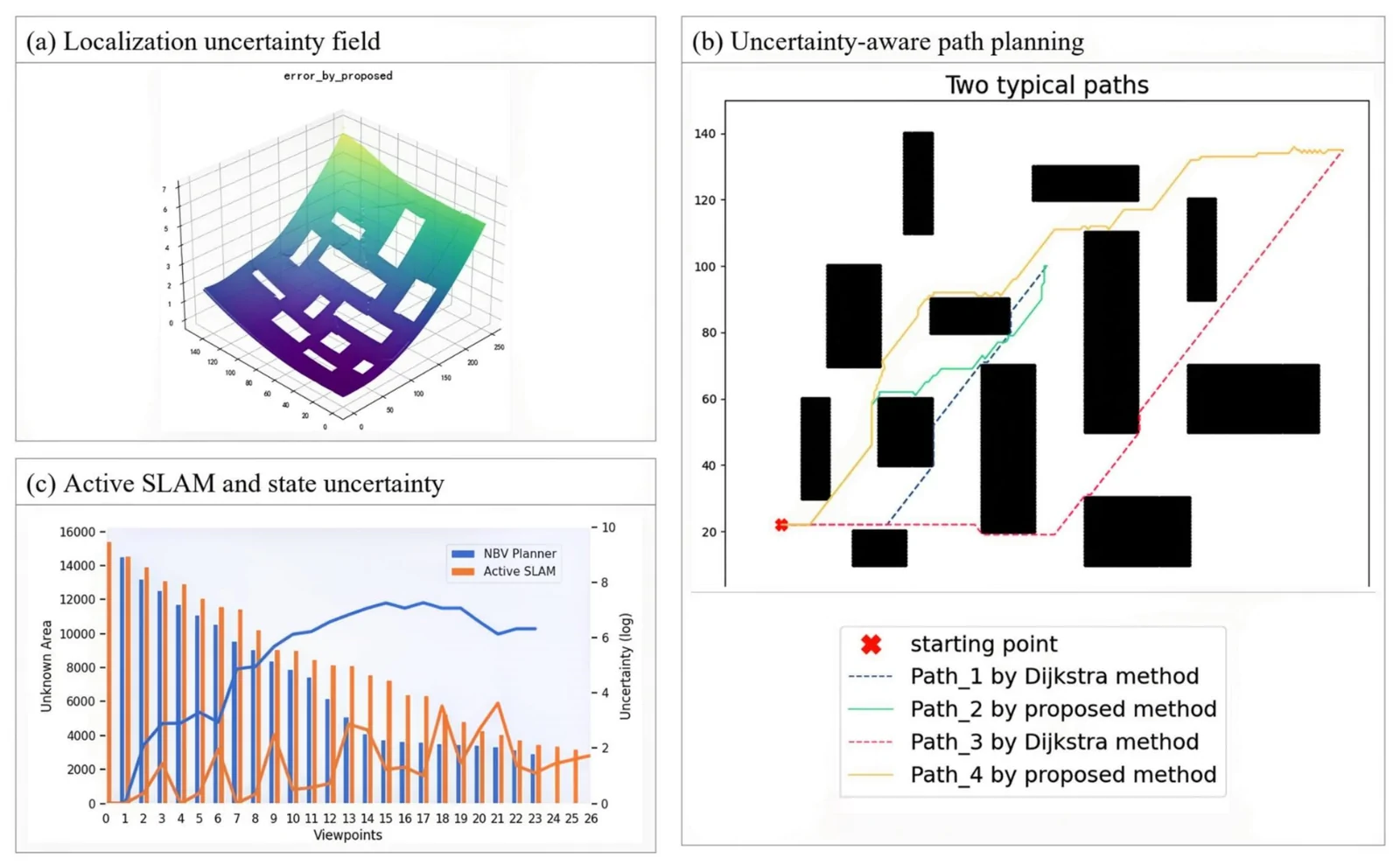
Propagation of localization uncertainty to mapping and planning with an active relocalization loop. Panels (**a**,**b**) are adapted and rearranged from Wang et al. [14], Figures 4 and 6, respectively; panel (**c**) is adapted and rearranged from Palomeras et al. [185], Figure 12. The source articles are distributed under the Creative Commons Attribution 4.0 International License (CC BY 4.0). In the source figure, the dark- and light-gray regions denote software-in-the-loop and hardware-in-the-loop, respectively; the blue and yellow fills distinguish functional blocks and mission-script elements as shown in the original diagram.

The synthesis is that localization uncertainty should be treated as a control variable for navigation behavior rather than a diagnostic displayed after planning. Simple covariance-based inflation is appropriate for low-cost systems with calibrated consistency; belief-space or active planning is justified when information quality varies spatially and the computing budget permits online evaluation. Their shared limitation is dependence on credible uncertainty estimates. Figure 15 shows the propagation into mapping and planning and the active-relocalization feedback [14,185]. The engineering recommendation is to define mission-specific protection and alarm limits and to validate the complete sensing-to-control response under injected degradation.

## 6. Trajectory Control, System Integration, and Typical Applications

Trajectory control transforms the risk-constrained trajectory generated in Section 5 into vehicle motion. It is also the stage where hydrodynamic disturbances, model errors, localization delay, and actuator limitations become concentrated. Control performance has system-level significance only when it is verified together with mission management, health monitoring, data recording, and manual takeover. Therefore, this section starts from the control interface. It then discusses classical and advanced methods, planning and control consistency, typical applications, and hierarchical evaluation. The emphasis is on how theoretical control performance is transformed into a maintainable and degradable mission closed loop.

### 6.1. Trajectory Tracking Problems and Control Interfaces

The technical question addressed in this section is how a planned path or trajectory should be translated into commands without losing the safety margins established by localization and planning. Guidance and interface options should be compared through accuracy, robustness, computational cost, environmental adaptability, failure modes, and deployment conditions. A hierarchical interface normally separates guidance, feedback control, and thrust allocation. Pure Pursuit and line-of-sight guidance are computationally light and effective for smooth transects, but small lookahead increases oscillation and large lookahead causes corner cutting. Their accuracy therefore depends on speed, curvature, current, and pose delay, and their deployment boundary is narrower in confined near-wall tasks than in open-water survey lines.

The interface must transmit curvature, velocity and acceleration limits, timestamps, validity intervals, uncertainty-dependent margins, and sensor-view requirements. Discrete waypoints alone force the controller to create its own smoothing and may invalidate collision clearance. Localization delay shifts the apparent tracking error, while delayed replanning can command a trajectory that is no longer valid. For visual or sonar inspection, body tracking and sensor line of sight must be coordinated so that standoff and incidence remain within the payload’s measurement envelope [186].

Control performance should be compared through lateral, depth, and heading error, high-error percentiles, settling and recovery time, peak input, saturation duration, energy per distance, and behavior under current, model mismatch, pose noise, and delay. Fish-cage simulation demonstrates that controller results are meaningful only relative to the vehicle dynamics, track geometry, and observation task [42]. Average error describes nominal accuracy; maximum deviation and recovery time describe robustness and collision relevance; computation and saturation describe deployability.

Simple guidance suits low-cost platforms and gentle paths; confined or high-current tasks require feasible references, delay compensation, and tighter thrust-allocation coupling. Ground-vehicle methods provide interface principles [187,188,189,190], but six-degree-of-freedom dynamics and current require underwater parameterization and validation [191,192,193].

### 6.2. Classical and Advanced Disturbance-Rejection Control Methods

The technical question addressed in this section is which controller provides the required tracking accuracy and disturbance rejection within the platform’s model, computation, and actuator limits. Proportional-integral-derivative (PID) control has low computational and maintenance cost and remains suitable for speed, heading, or depth channels over a bounded operating range. Feedforward, gain scheduling, filtering, and anti-windup improve performance, but multivariable coupling, parameter variation, strong current, and saturation remain characteristic failure modes [194]. PID is therefore an appropriate baseline and fallback, not a universal solution for tightly constrained maneuvering.

Backstepping, adaptive control, sliding-mode control, disturbance observers, and prescribed-performance methods use dynamic structure to improve robustness to nonlinearities, uncertain parameters, and bounded disturbances [182,183,195]. Sliding mode offers strong rejection but may cause chattering and actuator wear; adaptive methods reduce parameter sensitivity but require sufficient excitation; backstepping depends on model structure and measurable states. Their nominal accuracy can exceed fixed-gain control, but deployment requires verified disturbance bounds, state observability, sampling rate, and saturation handling [196,197,198].

Model predictive control (MPC) explicitly coordinates state, input, obstacle, and energy constraints and therefore aligns naturally with feasible local planning. Its limitations include online optimization cost, model mismatch, memory demand, and possible solver failure. Reduced-order models, warm starts, bounded horizons, terminal constraints, and a safe backup controller improve embedded feasibility. Tube and Gaussian process MPC extend constraint handling and model adaptation [199,200]. Prescribed-performance, higher-order sliding-mode, and adaptive fuzzy methods strengthen disturbance rejection [201,202,203,204]. Event-triggered and fault-tolerant controllers address communication or actuator limitations [205,206]. They should be compared by worst-case solve time, model dependence, saturation, and recovery rather than theoretical convergence alone.

**Figure 16:**
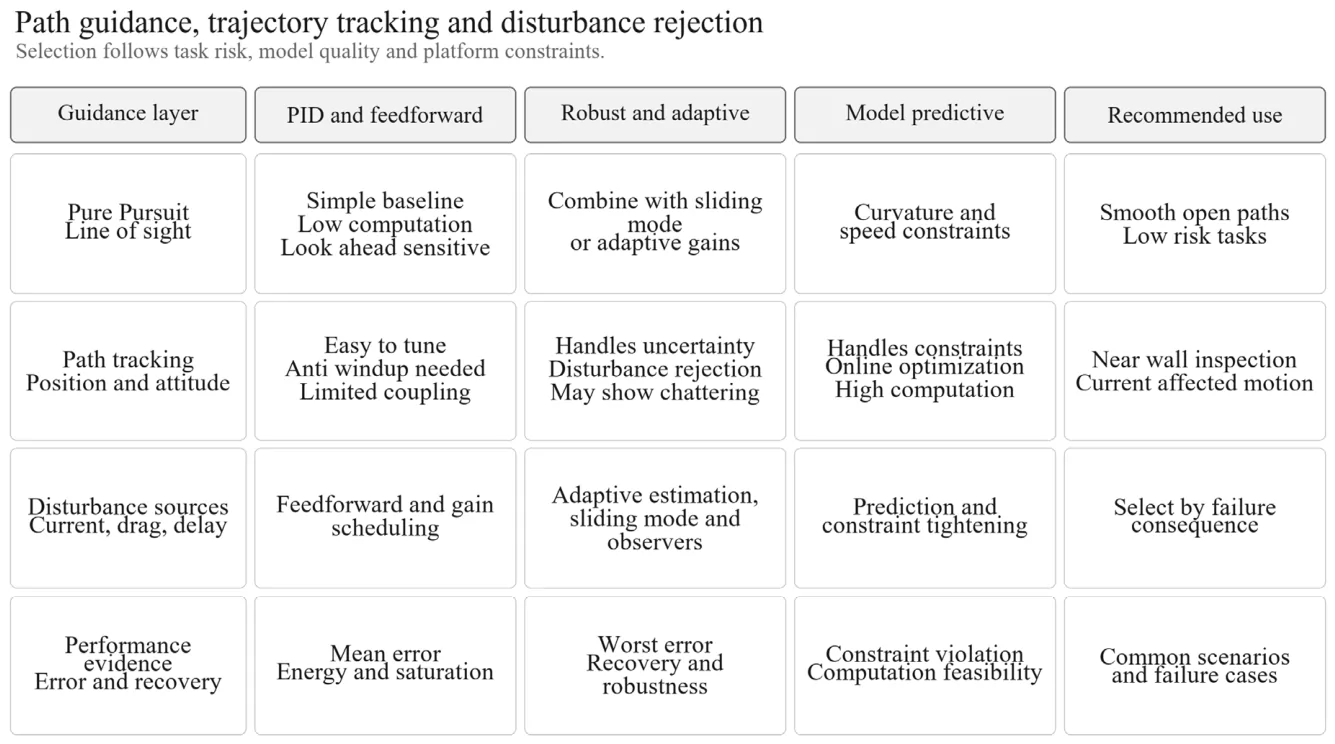
Comparison of path guidance, trajectory tracking, and representative disturbance-rejection controllers. Source: Created by the authors from underwater guidance and tracking evidence [182,183,194,195], MPC methods [199,200], robust and adaptive controllers [201,202,203,204], and event-triggered and fault-tolerant methods [205,206]; no source artwork is reproduced.

The comparative conclusion is to increase controller complexity only when the mission risk, model quality, localization credibility, and computing platform justify it. PID is preferred for maintainable low-risk operation; robust or adaptive methods for significant but bounded disturbance and parameter change; and MPC for constraint-intensive near-wall or multi-axis tasks with verified solver timing. Disturbance-observer and MPC studies emphasize disturbance bounds and solver constraints [207,208]. Backstepping and sliding-mode studies emphasize convergence, sideslip, and actuation limits [209,210,211,212]. Figure 16 summarizes these tradeoffs. The engineering recommendation is to retain a deterministic fallback, monitor saturation and estimator delay, and test the controller with the same uncertainty and fault cases used for planning [213].

### 6.3. Planning and Control Consistency and Safety Monitoring

The technical question addressed in this section is how to ensure that a risk-aware plan remains trackable and safe after localization delay, hydrodynamic disturbance, and actuator limitations are introduced. A trajectory lies within the platform’s executable domain only when curvature, acceleration, attitude rate, thrust allocation, stopping distance, and current compensation remain feasible. Reachable sets, dynamic windows, model predictive interfaces, or experimentally measured tracking-error envelopes can feed control capability back to planning. The total safety margin should combine localization uncertainty, map uncertainty, predicted obstacle motion, and expected tracking error rather than assigning each module an independent allowance.

A safety monitor independent of the nominal navigation algorithm should compare estimated state and subsystem health against mission limits. Depth, attitude, speed, clearance, battery, leakage, communication, localization credibility, computation delay, and control saturation represent different failure channels. Deceleration, hover, retreat, relocalization, surfacing, return, and buoy release should have explicit priorities and entry and exit conditions. Tethered systems additionally require tension and entanglement monitoring; untethered systems require autonomous termination when external communication is lost.

The synthesis is that planning-control consistency is a closed-loop property, not a planner or controller metric. Fault injection should cover camera occlusion, sonar multipath, DVL lock loss, IMU bias, map changes, thrust degradation, communication loss, and computational overload, while recording detection time, false alarms, error propagation, mode-switch stability, recovery, and minimum clearance. The limitation of conservative safety logic is reduced mission efficiency; the limitation of aggressive logic is silent margin erosion. The engineering recommendation is to tune thresholds from joint fault tests and to verify that every detected loss of credibility leads to a predictable and executable safe action.

### 6.4. Navigation System Integration and Typical Applications

The technical question addressed in this section is how the sensing-localization-planning-control chain should be integrated to produce reliable mission data rather than only an accurate trajectory. Water quality and ecological monitoring require repeatable spatial profiles, synchronized sensor response, stable speed, and position confidence [20]. Low-speed coverage, dwell at key points, and targeted reinspection improve data quality, but increase energy and current-exposure costs. The appropriate configuration depends on whether spatial coverage, temporal sampling, or rapid anomaly confirmation is the primary product.

Aquaculture and near-wall inspection place greater weight on standoff, viewing geometry, dynamic-organism rejection, and recoverability. Laser- and camera-assisted cage navigation, boundary inspection, and net observation show that geometric constraints, perception, and control must be co-designed [22,41,42]. Their transfer to fish ponds and confined inland structures is limited by shallow-water multipath, repeated walls, turbidity, and fish motion. Sonar improves environmental adaptability, vision improves fine defect detail, and multiaxis propulsion improves trackability, but each adds cost, calibration, and power demand.

**Figure 17:**
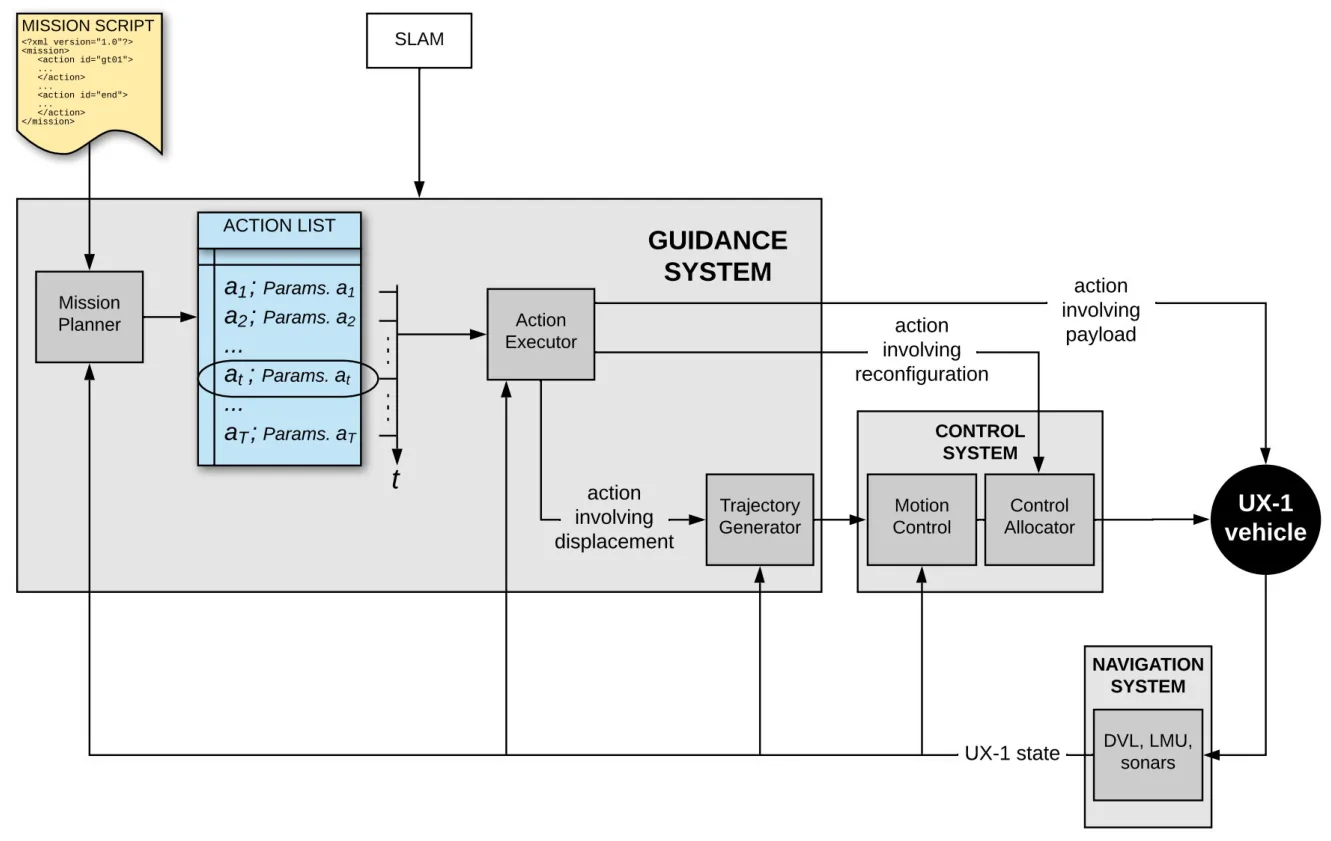
Mission planning, guidance, navigation, and control loop of the UX-1 underwater vehicle. Source: Reproduced from Ref. [37], Figure 5, under CC BY 4.0.

Critical hydraulic and confined facilities require high integrity because pose error affects both defect coordinates and collision risk [15,23]. Figure 17 shows how the UX-1 architecture links mission scripts, SLAM, planning, guidance, control, payload actions, and reconfiguration [37]. Its implication is causal: sensing quality conditions pose credibility, pose conditions mapping and motion, and control determines observation geometry. Mission records should retain raw evidence, pose uncertainty, sensor health, and repeat-inspection correspondence. Acceptance should assess mission success, data validity, intervention, and recovery, not subsystem accuracy alone.

### 6.5. Simulation Platforms, Datasets, and Unified Evaluation

The technical question addressed in this section is what evidence is sufficient to compare accuracy, robustness, computation, environmental adaptability, failure behavior, and deployment readiness. Simulation provides controlled testing of vehicle dynamics, current, sensors, communication, and faults, but its conclusions depend on model and parameter fidelity. Gazebo, UUV Simulator, Stonefish, and custom models are most useful when calibrated against bench or tank measurements. Experiments should progress from an ideal baseline to single degradation, interacting degradations, fault injection, and repeated statistical trials instead of relying on one successful trajectory.

Public datasets provide reproducible sensing and localization evidence at lower cost. AQUALOC, Tank, and ROV6D cover different visual, inertial, pressure, acoustic, and target-pose tasks [50,51,214], but do not fully represent inland turbidity variation, shallow-water multipath, dynamic fish, flow, fouling, or closed-loop control. Dataset performance therefore establishes algorithmic accuracy and partial robustness, not deployment readiness. Useful releases should include raw measurements, calibration, timestamps, reference trajectories, environmental metadata, failure events, evaluation scripts, and computing conditions.

Evidence should be interpreted hierarchically. Algorithms establish estimation, planning, or control behavior; subsystem tests add timing, memory, power, availability, and recovery; mission tests add coverage, data validity, intervention, energy, and success. Table 7 retains the minimum fields and Table S4 provides the detailed evidence. Simulation, replay, hardware-in-the-loop, tanks, and repeated field trials provide progressively stronger but noninterchangeable evidence.

Quantitative reporting should distinguish sensor, estimator, planner, controller, and mission timing. Examples include 100 Hz IMU and 1 Hz DVL [36], 200 Hz IMU with 0.5 Hz USBL and 1 Hz DVL [82], and 20 Hz camera with 5 Hz pressure in AQUALOC [16]. Unreported control frequency, sensing range, DVL loss distance, power, or worst-case latency must remain unreported.

Unreported visual range, DVL loss distance, control frequency, embedded power, and worst-case replanning latency are not inferred.

### 6.6. Mission-Oriented System Configuration and Engineering Maturity

Engineering maturity combines mission configuration with evidence. Recoverable pond monitoring can use a compact platform and simple localization and guidance; mapping generally requires DVL, current compensation, relay, and energy-aware planning; critical inspection adds heterogeneous redundancy, integrity monitoring, multiaxis propulsion, constrained control, and fail-safe recovery [215].

**Figure 18:**
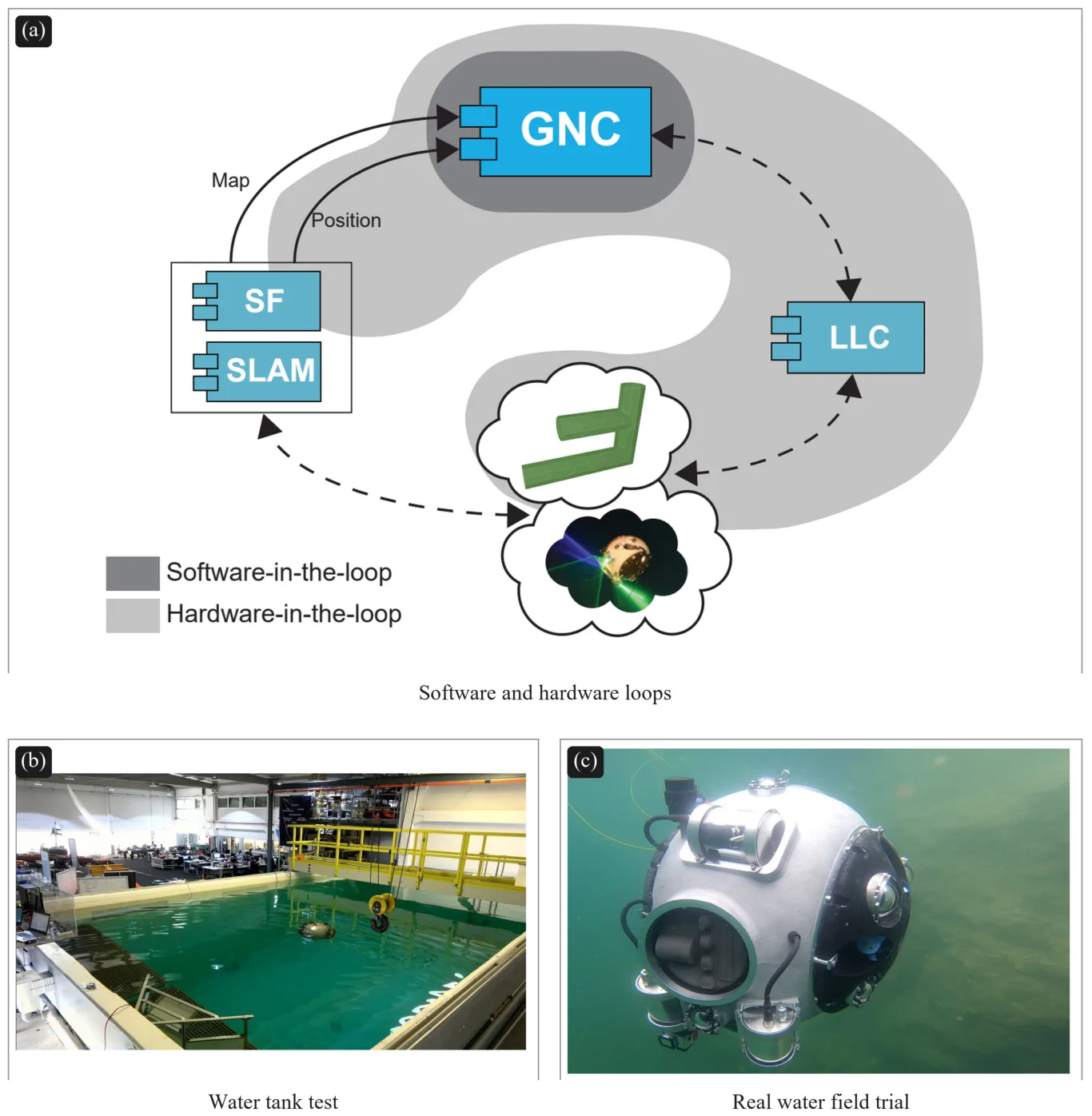
Progressive validation of underwater robot navigation from software and hardware loops to tank and real-water trials. Source: Panels (**a**–**c**) adapted from Ref. [37], Figures 7, 10, and 1, respectively, under CC BY 4.0. The panels were cropped and rearranged by the authors. Panel (**a**) distinguishes software-in-the-loop and hardware-in-the-loop; panels (**b**,**c**) are illustrative validation scenes, and arrows retain their original meaning.

Maturity progresses from interface and bench tests through software and hardware loops, tanks, representative waters, and repeated cross-season trials. Each level adds timing, dynamics, turbidity, multipath, current, recovery, maintenance, and cost evidence. Figure 18 summarizes this progression.

The remaining bottleneck is incomplete transfer of credibility and constraints across SLPC. Systems should establish an interpretable, maintainable, degradable minimum loop before adding sensors or optimization. Advancement should depend on mission success, high-error behavior, worst-case computation, availability, recovery, data validity, energy, intervention, and life-cycle cost.

## 7. Limitations and Research Directions

The preceding synthesis identifies limitations that recur across sensing, localization, planning, control, and system validation. This chapter separates these limitations from the conclusions. It examines each one through four questions: what the problem is, why it occurs in inland waters, which part of the navigation chain it affects, and which mitigation routes warrant further investigation. Unless explicitly supported by field evidence, the mitigation measures discussed below are presented as research directions rather than validated engineering conclusions.

### 7.1. Sensing Degradation and Insufficient Observability

The first limitation is that environmental degradation can reduce both measurement quality and state observability. Turbidity, light attenuation, suspended particles, repetitive boundaries, shallow-water acoustic multipath, dynamic organisms, and loss of DVL lock do not merely add independent noise. Visual degradation can remove geometric constraints or create biased associations [16,52]. DVL loss and inertial disturbances can weaken velocity observability [36,104,119]. Sonar systems are vulnerable to ambiguous associations, poor overlap, and geometric degeneracy [54,55,56,64]. Reviews of underwater fusion and operational localization show that correlated environmental effects may degrade several sensors simultaneously [109,118].

This limitation originates from the mismatch between nominal sensor assumptions and the spatially and temporally varying conditions of inland waters. Reduced feature repeatability, ambiguous echoes, beam invalidity, or poorly excited motion first decrease the information available to the estimator. The resulting loss of observability then increases pose drift, distorts map registration, narrows the planner’s reliable free space, and may finally erode tracking clearance even when the controller itself remains stable. Average image quality or nominal sensor precision therefore cannot establish navigation credibility.

A research direction is to combine task-oriented front-end quality metrics, online observability tests, heterogeneous sensing, and explicit degraded modes. Visual feature distribution and front-end residuals should be checked before fusion [46,49,53,58]. Sonar association and scan geometry require separate consistency tests [54,55,56,64]. DVL beam status and innovation statistics support rejection of abnormal velocity or acoustic updates [82,83,87,89]. Calibration and timing quality should also be monitored before measurements enter tightly coupled estimation [69,117]. These measures may support early rejection, sensor switching, speed reduction, or relocalization, but their thresholds and false-alarm behavior still require validation under combined degradation. The engineering implication is to configure redundancy around independent failure mechanisms rather than sensor count alone.

### 7.2. Model Mismatch and Credibility Limitations in Multisensor Localization

The second limitation concerns model mismatch and the credibility of the fused state. Filters and optimization methods generally assume calibrated extrinsics, synchronized timestamps, specified noise distributions, valid associations, and sufficiently exciting motion. Mounting changes, correlated noise, persistent bias, delayed measurements, and domain shift violate these assumptions. Tightly coupled acoustic, visual, inertial, pressure, and DVL systems retain raw cross-sensor constraints [69,76,77,79,81]. However, one biased factor can propagate across several states. Modular filtering and robust graph methods can isolate or downweight faults more directly, but may discard useful correlations [82,83,84,87,89].

The main consequence is inconsistency between reported covariance and actual position error. A pose estimate may appear smooth and accurate while its protection information is overconfident. This affects map occupancy, obstacle inflation, active relocalization, and safety-state decisions because downstream modules cannot distinguish a small credible error from a larger unreported one. Learning-assisted switching and fault diagnosis can improve adaptation [91,92,93,94]. However, domain shift and uncalibrated confidence introduce an additional failure channel, so confidence assessment requires independent calibration [120,121].

Potential mitigation should be treated as a research direction centered on integrity rather than trajectory error alone. Candidate elements include residual and information-matrix monitoring, adaptive noise models, fault isolation, estimator switching, bounded sliding-window optimization, protection levels, and independent consistency checks for learned outputs. Future experiments should report detection latency, false alarms, covariance consistency, recovery time, and failure containment in addition to root-mean-square error [82,91,92,109,118]. Until these indicators are validated, higher nominal accuracy should not be interpreted as higher operational trustworthiness.

### 7.3. Coupling Limitations Between Risk-Aware Planning and Motion Control

The third limitation is the incomplete coupling of localization risk, map uncertainty, trajectory feasibility, and control authority. Many planning studies assume an exact pose, a static map, and perfect path tracking, while control studies commonly receive a geometrically feasible reference without the uncertainty and sensing constraints that shaped it. A route can be collision-free in the planning model but unsafe under current or moving-obstacle uncertainty [172,173,174,175]. Pose delay and localization uncertainty can further invalidate the planned clearance [181,216]. Actuator constraints and solver timing must therefore be included when trajectory feasibility is assessed [199,206].

The propagation mechanism is sequential and bidirectional. Sensing degradation weakens localization; pose uncertainty broadens or misaligns mapped obstacles; planning based on that map may underestimate clearance or generate excessive conservatism; and tracking error, solver delay, and thrust limits further reduce the remaining margin. Conversely, platform reachability, stopping distance, and expected tracking error determine which paths are genuinely safe. Treating these quantities in separate modules obscures the combined risk and prevents reliable diagnosis of mission failure.

A research direction is to establish explicit uncertainty and feasibility interfaces among localization, mapping, planning, and control. Covariance-based obstacle inflation, chance constraints, belief-space planning, active localization, reachable sets, safety filters, and model-predictive interfaces provide candidate mechanisms [181,199,200,216,217]. Their practical value remains conditional on credible uncertainty, bounded computation, and experimentally identified tracking envelopes. These approaches should therefore be tested against deterministic fallback planning and control under the same injected sensor, map, timing, and actuator faults.

The engineering implication is to define a mission-level state machine. Normal credibility permits efficiency-oriented motion; reduced credibility enlarges margins, reduces speed, and favors informative trajectories; an exceeded alarm limit triggers relocalization, retreat, surfacing, return, or termination. This structure does not eliminate uncertainty, but it makes its consequences observable and links each risk level to an executable response.

### 7.4. Resource, Communication, Energy, and Low-Cost Platform Constraints

The fourth limitation arises because advanced perception, graph optimization, learned models, and constrained planning compete with propulsion, lighting, sonar, and communication for limited computation and energy. Low-cost platforms also have tighter volume, thermal, waterproofing, payload, and maintenance constraints. Acoustic links provide limited bandwidth and may suffer from latency and shallow-water multipath [44]. Optical links require favorable visibility and alignment [44]. Tethers support high-bandwidth supervision but add drag and entanglement risk [23,124].

Resource shortage affects the complete closed loop rather than only software speed. Computational overload delays sensing and localization, makes the map stale, shortens replanning time, and increases tracking and collision risk. Energy depletion restricts illumination, acoustic operation, current rejection, return range, and fault recovery. Communication interruption removes supervision and high-bandwidth data transfer, while thermal throttling or memory pressure may make nominal real-time performance unsustainable. These effects are especially consequential when low hardware cost is achieved by removing independent sensing or recovery functions.

A research direction is to develop resource-aware navigation with measured worst-case latency, memory, power, thermal behavior, and task-shedding thresholds. Deterministic filtering, bounded optimization windows, adaptive update rates, edge processing, energy-aware planning, and independent fallback control are plausible mitigation routes, but their effectiveness must be verified on the target embedded platform rather than inferred from desktop execution. The deployment recommendation is to preserve attitude, depth, basic obstacle avoidance, return, and logging before optional mapping or semantic functions, and to test the minimum safe loop under low battery, communication loss, and computational overload.

### 7.5. Dataset, Evaluation Standardization, and Real-Water Validation Gaps

The fifth limitation is the insufficiency of comparable datasets, unified evaluation standards, and repeated real-water evidence. Existing public datasets and experiments cover useful visual, inertial, pressure, acoustic, or target-pose tasks. However, they do not jointly represent the turbidity variation, shallow-water multipath, dynamic organisms, flow, fouling, communication interruption, and closed-loop recovery required for inland deployment [53,62,63,214]. Studies also use different sensors, ground-truth systems, maps, mission scales, computing platforms, and metrics. Numerical results therefore cannot be treated as a unified benchmark.

This evidence gap limits both scientific comparison and engineering transfer. Missing environmental metadata, calibration records, failure cases, repeated trials, and computing conditions make it difficult to determine whether an improvement comes from the method, hardware, scenario, or evaluation design. Simulation and dataset replay can establish algorithmic behavior, but they cannot alone demonstrate closed-loop safety, maintenance stability, recovery performance, or cross-season robustness. Consequently, reported accuracy may define only a narrow validation boundary rather than deployment maturity.

A research direction is to establish hierarchical inland-water benchmarks that share scenario descriptions, raw measurements, calibration, synchronized ground truth, environmental parameters, fault definitions, evaluation scripts, and computing conditions. Public datasets support reproducible replay and algorithm comparison [50,51,214]. Confined-environment experiments and modular system studies support the next stages of hardware-in-the-loop and tank validation [37,148]. Evidence should then progress to representative water bodies, repeated trials, and cross-season operation. Minimum reporting should include high-percentile errors, latency, resource use, sensor availability, fault detection and recovery, mission success, data validity, intervention, and energy. This framework remains a proposed validation direction until shared protocols and multi-site evidence are available.

### 7.6. Integrated Mitigation Priorities and Phased Validation

Across the five limitation classes, mitigation should follow the causal order of the navigation chain. Short-term work should first make degradation observable through calibration records, quality indicators, fault injection, and reproducible baselines. Medium-term work should verify uncertainty propagation, degraded modes, replanning, fallback control, and recovery in representative fish ponds, rivers, reservoirs, or hydraulic structures. Longer-term work should examine repeated cross-season operation, maintenance, remote supervision, standardized task data, and life-cycle cost. Advancing to a later stage is justified only when both normal and degraded behavior pass the preceding evidence level.

**Figure 19:**
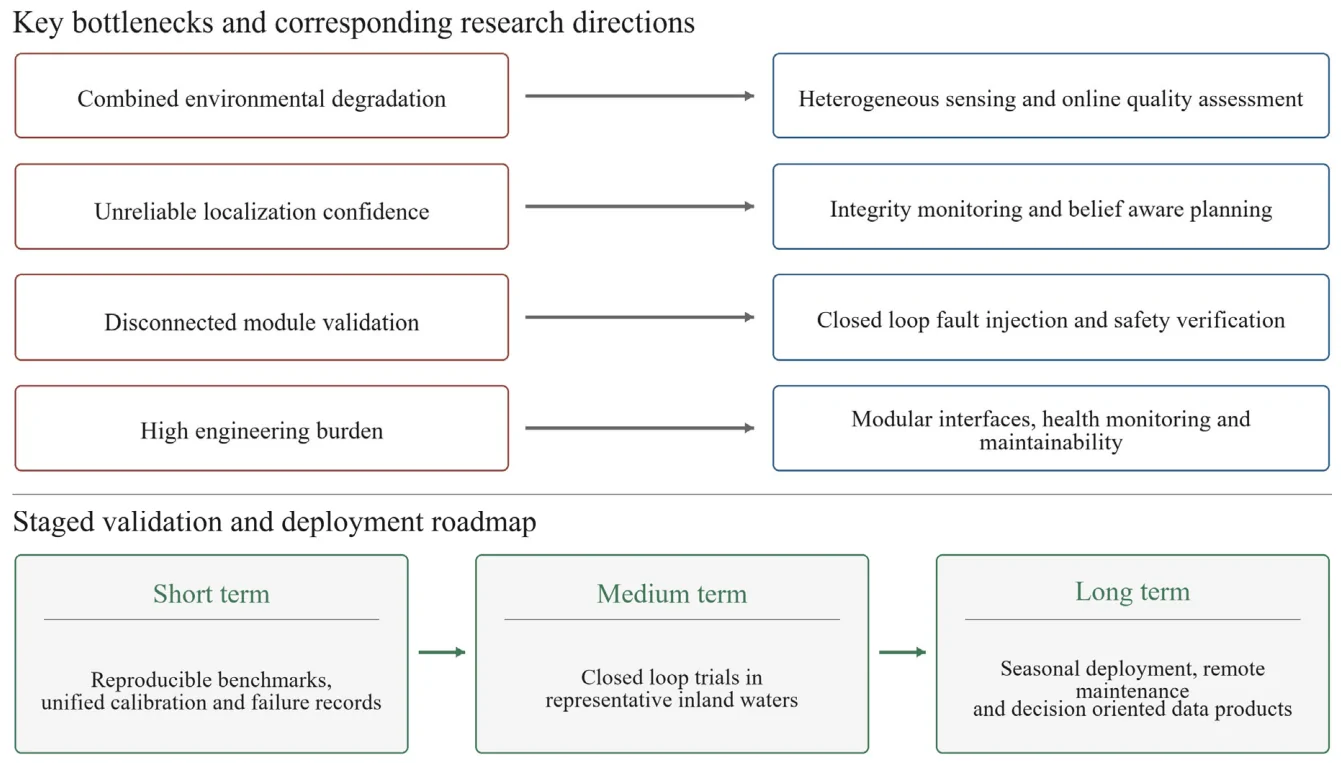
Bottlenecks, research directions, and staged validation roadmap for underwater robot navigation in inland waters. Source: Redrawn and synthesized by the authors from discussions of underwater navigation, active localization, system closed loops, and engineering validation in Refs. [3,4,5,37,109,148,217]; no source artwork is reproduced.

Table 8 summarizes the five limitation classes and candidate mitigation directions, while Figure 19 maps them to staged validation. These directions are research hypotheses, not established performance claims.

## 8. Conclusions

Underwater robot navigation in inland waters should be organized as a coupled sensing, localization, planning, and control chain whose behavior is conditioned by mission risk. The reviewed evidence indicates that no single sensor, estimator, planner, controller, or platform configuration dominates across turbidity, shallow-water multipath, confined geometry, dynamic organisms, current, communication limits, and cost constraints. Effective configurations instead combine complementary observations, explicit localization credibility, risk-aware and dynamically feasible planning, resource-aware control, and predictable degraded operation.

The principal contribution of this review is not a universal ranking of methods. Instead, it integrates how environmental degradation affects sensing and how sensing quality determines localization credibility. It further traces how uncertainty propagates into mapping, path selection, trajectory feasibility, and control safety. For near-term deployment, a modular, diagnosable, and gracefully degradable closed loop is more defensible than adding complexity without corresponding evidence. Reliable operation should be judged by mission completion, data validity, high-error behavior, computation and energy limits, fault detection, recovery, and maintainability within stated application boundaries.

## Figures and Tables

**Table 1:** Conditional technical comparison between this review and the collective emphasis of existing related reviews; the comparison indicates analytical scope rather than a unified performance benchmark. Data source: representative English-language reviews cited in this article; no individual publication is graded.

Technical Comparison Dimension	Typical Emphasis in Existing Related Reviews	Analytical Treatment in This Review
Inland-water specificity	Evidence is commonly organized around open-sea surveys, deep-water AUVs, offshore structures, or general underwater navigation, with limited differentiation among lakes, reservoirs, rivers, fish ponds, and confined hydraulic facilities.	Environmental mechanisms and inspection tasks are linked explicitly to sensing availability, localization observability, planning constraints, and control margins in shallow, turbid, boundary-dense inland waters.
Coupled sensing degradation	Visual, acoustic, inertial, velocity, and depth sensing are often reviewed by modality or algorithm family, mainly according to nominal accuracy and operating principle.	Sensor complementarity is compared under turbidity, acoustic multipath, repetitive geometry, biological interference, DVL lock loss, synchronization error, and calibration drift, with attention to simultaneous degradation.
Localization credibility	Localization performance is generally represented by trajectory error, drift, or the structure of filtering and SLAM algorithms.	Accuracy is considered together with consistency, observability, anomaly rejection, degradation detection, integrity monitoring, availability, and the confidence needed for downstream decisions.
Uncertainty propagation	Localization, mapping, planning, and control are frequently treated as adjacent but largely independent technical modules.	Pose covariance, sensor health, and map confidence are traced through obstacle inflation, collision probability, route selection, active relocalization, tracking margins, and inspection data validity.
Risk-aware planning and control	Planning comparisons commonly emphasize path length, convergence, or obstacle avoidance while assuming sufficiently accurate pose and map information.	Path cost and executable motion are assessed jointly using environmental risk, localization uncertainty, energy margin, vehicle dynamics, replanning latency, safety monitoring, and degraded operation requirements.
Embedded computing	Advanced fusion and planning methods are mainly compared by algorithmic performance; onboard processing, memory, power, and implementation constraints are not always reported on a common basis.	Estimator and planner selection is interpreted against real-time feasibility, fusion complexity, update rate, embedded CPU or GPU demand, memory and power constraints, and the payload limits of low-cost platforms.
Engineering validation	Results are synthesized across simulation, public datasets, tanks, and field trials, although differences in ground truth, duration, disturbances, and failure injection limit direct comparison.	Evidence is stratified by validation environment and maturity, and interpreted using ground-truth quality, trial duration, repeatability, fault recovery, mission success, and long-term deployment relevance.
Configuration guidance	Conclusions commonly summarize individual sensor, localization, planning, or platform trends.	The synthesis produces task-conditioned links among environment, sensor combination, fusion framework, uncertainty interface, planning and control strategy, computing resources, and reliability verification route.

**Table 3:** Navigation performance metrics and their correspondence with SLPC stages. Data source: the metric framework in Section 2.5 and Section 6, with conditioned engineering examples from Refs. [16,36]. Reported values remain test-specific and are not a unified benchmark.

Metric Category	Recommended Metric	Unit or Expression	Main Meaning	Applicable Task
Sensing	Effective detection range and observation coverage	m; deg; %	Range within which obstacle, boundary, or target information can be stably obtained	Obstacle avoidance and structural inspection
Sensing	Missed detection rate, false detection rate, and processing latency	%; s	Observation reliability, update timeliness, and degree of degradation	Dynamic obstacle avoidance and target detection
Localization	Absolute trajectory error (ATE)	m/RMSE	Overall deviation of the estimated trajectory from the reference trajectory	Mapping and long-voyage inspection
Localization	Relative pose error (RPE)	m, deg/time interval	Translational and rotational drift over a short time interval	Odometry and trajectory tracking
Localization	Availability, consistency, and relocalization time	%; normalized estimation error squared (NEES); normalized innovation squared (NIS); s	Proportion of trustworthy pose outputs and abnormal recovery capability	Long-term autonomy and repeated reinspection
Planning	Path cost and planning/replanning time	m; s; J	Voyage length, computational cost, and energy efficiency	Point-to-point, coverage, and dynamic replanning
Planning	Minimum obstacle distance and collision risk	m/confidence lower bound, probability	Safety margin considering localization and sensing uncertainty	Near-wall inspection and dynamic obstacle avoidance
Control	Trajectory tracking error, settling time, and overshoot	m; deg; s; %	Trajectory executability, dynamic response, and stability	Narrow channels and near-wall tracking
Control	Input smoothness, saturation ratio, and disturbance-rejection recovery	Rate of change, %, s	Actuator load and recovery capability under current disturbances	Navigation in flow fields and degraded operation under faults
Mission Level	Coverage, energy consumption, and mission success rate	%; Wh; J	End-to-end mission completion capability of the SLPC closed loop	All autonomous inspection tasks
Conditioned engineering examples	IMU 100 Hz; DVL 1 Hz; camera 20 Hz; pressure 5 Hz	Hz; test-specific	Update rates are not equivalent to effective range, estimator latency, or closed-loop availability	Report scenario, sensor configuration, ground truth, and whether the value is measured or configured [16,36]

**Table 4:** Conditional comparison of fusion-localization method families for inland-water missions. Evidence groups comprise general architectures and reviews [16,17,18,19,109,118] and datasets and sensing evidence [50,51,52,123]. Additional groups cover visual and acoustic localization [46,47,48,49,53,54,55,56,58,62,63,64,65,69] and fusion, robustness, and degraded-mode methods [76,77,78,79,80,81,82,83,84,85,86,87,88,89,90,91,92,93,94,104,105,106,107,108,117,122]. The comparison is conditional; detailed sensor configurations and source-reported computing evidence are retained in Tables S1 and S2.

Main Limitation	Recommended Inland-Water Use	Computational Profile	Main Advantage	Method Family
Limited smoothing and loop closure use	Low-cost, high-rate localization	Low to moderate; bounded state	Low-latency recursive update	Recursive filtering
Solver latency and erroneous-factor propagation	Long-range or high-accuracy missions	Moderate to high; grows with window and active factors	Uses multi-time-step constraints and robust estimation	Sliding-window or factor-graph optimization
Sensitive to low visibility and weak texture	Structured scenes with usable visibility	Moderate to high; front-end and mapping costs	Provides local geometry without acoustic infrastructure	Visual–inertial localization
Calibration and embedded implementation burden	Turbid, near-wall, or safety-relevant missions	High; synchronization and joint optimization	Jointly exploits complementary observations	Tightly coupled multimodal fusion
Requires validated thresholds and backup observations	High-consequence or long-duration missions	Moderate to high; monitoring and mode switching	Outputs credibility and supports graceful degradation	Integrity-aware and fault-tolerant fusion

**Table 5:** Conditional platform configurations for representative inland-water tasks. Evidence groups: underwater-platform and mission reviews [1,2,3,15,17,18,19,20,44,45]; modular vehicle and software systems [22,23,112,117,147,148]; and inspection and mission-payload applications [129,130,131,132,133,149,150,151,152]. The comparison is conditional; detailed platform evidence is retained in Table S3.

Verification Priority	Core Sensing and Payload	Platform Priority	Typical Mission
Obstacle avoidance, sampling position, and rapid recovery	IMU, depth, camera or sonar, and water quality sensors	Small ROV or low-speed AUV; low-disturbance maneuvering	Fish pond water quality and aquaculture inspection
Current compensation, moving obstacles, and DVL outage	IMU, DVL, depth, and forward-looking sonar	Streamlined AUV or surface-underwater cooperation	River cross-section and revetment inspection
Long-range drift, coverage, energy, and communication	INS, DVL, bathymetric sonar, and profiling payload	Endurance AUV with optional surface support	Lake and reservoir mapping
Near-wall control, collision, faults, and manual takeover	Vision-sonar redundancy, IMU, DVL, and inspection payload	Maneuverable ROV or high-reliability AUV	Dam, pier, and confined-facility inspection

**Table 6:** Capability boundaries and applicable conditions of path-planning methods. Evidence groups: graph, sampling, and review evidence [11,12,13,40,184]; mission and energy-aware planning [153,154,155]; intelligent optimization and reinforcement learning [164,165,166,167,168]; and underwater local obstacle avoidance [172,173,174]. The entries depend on map quality, dynamic-obstacle modeling, computing resources, and control interfaces and should not be used as a unified performance ranking.

Method Category	Main Advantages	Main Limitations	Applicable Tasks	Uncertainty Interface
Dijkstra/A* graph search	Stable and interpretable; can obtain discrete globally optimal or near-optimal paths	Depends on map resolution; high computational load in three dimensions	Regular grids, point-to-point tasks, and global navigation strips	Cost map inflation and risk weights
D*/incremental search	Reuses historical search results and is suitable for local map updates	Benefits decrease under frequent large-scale changes	Online replanning and new obstacles at boundaries	Updates occupancy probability and safety boundaries
PRM/RRT/RRT*	Suitable for continuous high-dimensional spaces and complex obstacles	Problems with narrow-passage efficiency, stochastic stability, and postprocessing	Complex structures and planning with kinematic constraints	Probabilistic collision screening and belief sampling
Swarm intelligence/evolutionary optimization	Readily handles multiobjective and nonconvex costs	Sensitive to parameters; insufficient worst-case delay and repeatability	Offline navigation strips and energy-consumption and flow-field optimization	Risk terms enter the fitness function
Learning-based/hybrid planning	Can use dynamic prediction and complex environmental representation	Insufficient handling of domain shift, interpretability, and safety guarantees	Dynamic obstacle avoidance and sensing quality prediction	Confidence, chance constraints, and safety filtering
Timing evidence and boundary	A confined-tunnel SIL case reported 1.3 s collision-detection delay and 2.3 s path-planning time	No common real-water replanning benchmark; delay depends on map, checker, solver, and platform	Use as a traceable case only, not as a cross-study ranking [37]	Expose delay to risk margins, speed limits, and fallback actions

Note: Sources are grouped by graph, sampling, and review evidence [11,12,13,40,184], mission and energy-aware planning [153,154,155], intelligent optimization and reinforcement learning [164,165,166,167,168], and local obstacle avoidance [172,173,174]. For safety-critical missions, a hybrid framework should retain explicit constraints and fallback strategies.

**Table 7:** Conditional minimum evaluation framework for the navigation closed loop. Data source: studies and datasets discussed in Section 6.1, Section 6.2, Section 6.3, Section 6.4 and Section 6.5, including Refs. [37,50,51,214]. Evidence levels are not interchangeable; fields not traceably reported in the cited source remain Not reported.

Interpretation Boundary	Minimum Evidence	Core Metrics	Evaluation Level
Report visibility, geometry, multipath, and sensor outages	Dataset replay and tank ground truth	ATE, RPE, consistency, availability, relocalization time	Sensing and localization
Report map dynamics and localization uncertainty	Monte Carlo simulation and hardware-in-the-loop testing	Success rate, collision risk, replanning latency, energy margin	Planning
Report flow, delay, and actuator degradation	Dynamic simulation and bench or tank tests	Tracking error, settling time, saturation, energy use	Control
Report duration, failures, recovery, and data validity	Repeated deployments in representative real waters	Coverage, missed detection, repeat arrival, mission success, maintenance	System and mission
Resource and timing traceability	Report sensor rates, estimator and controller update rates, planning latency, CPU/GPU, memory, power, and test condition	Measured value with source; otherwise Not reported	Simulation, hardware-in-the-loop, tank, and real-water evidence must remain separate

**Table 8:** Major bottlenecks, existing boundaries, and potential breakthrough directions. Evidence groups: foundational navigation reviews [3,4,5], underwater sensor-fusion synthesis [109], confined-system validation [37,148], and active SLAM research [217]. The breakthrough directions are research hypotheses requiring validation, not established performance claims.

Key Bottlenecks	Existing Research Boundaries	Potential Breakthrough Directions	Validation Priorities
Sensing degradation and insufficient observability	Turbidity, multipath, repetitive geometry, dynamic organisms, and DVL lock loss can remove or bias constraints.	Task-oriented quality assessment, online observability monitoring, heterogeneous redundancy, and explicit degraded modes	Constraint availability, false alarms, missed degradation, drift growth, and recovery time
Model mismatch and insufficient localization credibility	Calibration drift, timing offsets, correlated noise, biased associations, and domain shift can make uncertainty estimates inconsistent.	Residual and information monitoring, adaptive noise models, fault isolation, protection levels, and independent confidence checks	Covariance consistency, detection latency, integrity risk, failure containment, and relocalization success
Incomplete coupling of planning and control	Pose and map uncertainty, tracking error, solver delay, and actuator limits are often evaluated separately.	Uncertainty-aware obstacle inflation, chance constraints, belief-space planning, reachable-set feedback, and safety filters	Closed-loop clearance, high-percentile tracking error, replanning latency, saturation, and safe completion
Resource, communication, energy, and low-cost platform constraints	Computation, memory, thermal capacity, power, bandwidth, payload, and maintenance resources are limited.	Bounded optimization, adaptive update rates, task shedding, energy-aware planning, and independent fallback control	Worst-case latency, memory, power, thermal throttling, communication loss behavior, and return margin
Dataset, evaluation-standard, and real-water validation gaps	Datasets, ground truth, scenarios, computing platforms, fault definitions, and metrics remain heterogeneous.	Hierarchical benchmarks with shared metadata, fault injection, repeated trials, and cross-season field validation	Reproducibility, data validity, mission success, intervention, recovery, maintenance, and life-cycle cost

## Data Availability

No new data were created or analyzed in this review. Data sharing is not applicable to this article.

## Supplementary Materials

The following supporting information can be downloaded at: https://www.mdpi.com/article/10.3390/s26185822/s1, Table S1. Detailed mission-oriented fusion-localization and sensor configurations; Table S2. Source-reported computational evidence and review-synthesized embedded applicability; Table S3. Detailed platform configurations for representative inland-water missions; Table S4. Detailed hierarchical metrics, degradation modes, and evidence recommendations.
