# Supplementary material for: Multisensor Localization and Risk-Aware Navigation for Underwater Robots in Turbid and Confined Inland Waters: A Review
Source: Sensors (Basel). 2026 Sep 14;26(18):5822. doi: 10.3390/s26185822 (PMC13611597; doi:10.3390/s26185822)
Supplement: Supplementary file 1 [file sensors-26-05822-s001.zip › sensors-4504825-supplementary.pdf]

Supplementary Materials: Detailed Evidence for Tables 4, 5, and 7

These supplementary tables preserve detailed algorithm, sensor, platform, parameter, and validation information removed from the main manuscript for readability. Reference numbers correspond to the final revised manuscript and its current 217-entry reference list. NR denotes not reported in the cited source.

Table S1. Detailed mission-oriented fusion-localization and sensor configurations.

Method and Configuration Type	Applicable Missions and Environments	Recommended Sensor Combination	Fusion Framework	Configuration Focus
Basic Real-Time Localization	Clear-water, short-range, and low-cost inspection	Camera, IMU, and depth meter	Extended Kalman filtering or lightweight sliding window	Monitor image quality and visual tracking status, and rely on inertial and depth information to maintain short-term continuity when vision fails
Enhanced Localization for Low Visibility	Turbid water, weak light, and near-wall structural inspection	Imaging sonar, IMU, and depth meter	Filtering estimation or sonar-inertial local optimization	Evaluate sonar matching quality and geometric observability, and suppress shallow-water multipath and mismatches in repeated structures
Combined Localization for Medium- and Long-Range Missions	Measurable pool bottom, continuous navigation, and coverage inspection	IMU, DVL, and depth meter, with optional vision or sonar	Filtering propagation and sliding-window correction	Explicitly identify DVL loss of lock and switch the noise model to limit the accumulation of velocity errors in the position state
High-Precision Localization with Absolute Constraints	Inspection of dams, gates, and critical facilities	IMU, DVL, depth meter, and USBL or LBL	Robust filtering or factor graph optimization	Use absolute position to suppress long-term drift while handling acoustic occlusion, multipath, base-station deployment errors, and abnormal measurements
Multimodal Robust Localization	Substantial transparency variation and frequent environmental degradation	Vision, sonar, IMU, DVL, and depth meter	Tightly coupled sliding window or factor graph	Strengthen time synchronization, extrinsic-parameter calibration, robust kernels, and measurement-quality weighting to prevent erroneous constraints from propagating to the global state
Safety-Critical Fault-Tolerant Localization	Long-term autonomous operation and high-consequence missions	Multimodal combination and backup absolute position information	Main estimator, integrity monitoring, and mode switching	Output pose, credibility, valid sensors, and degradation level, and coordinate deceleration, relocalization, surfacing, or return strategies

Data source: Summarized from Refs. [16–19,46–56,58,62–65,69,77–95,105–110,118–119,122–123]. The configurations are conditional recommendations rather than results from a unified benchmark.

Table S2. Source-reported computational evidence and review-synthesized embedded applicability.

Method and representative evidence	Reported computing platform	Reported real-time or rate evidence	Reported memory or computational burden	Embedded applicability
Recursive filtering: Lie-group EKF [87]; event-triggered nonlinear filter [90]	CPU: NR; GPU: NR; clock: NR	Ref. [87]: INS and DVL at 10 Hz in simulation. Ref. [90]: INS at 100 Hz and GPS at 1 Hz. Execution time: NR.	Memory: NR. Source-level burden: Ref. [90] notes that iterative robust estimation increases complexity; no measured memory or latency is given.	Review synthesis: most suitable starting point for Nano-class hardware when state size and diagnostic logic remain bounded; verify worst-case update latency on target hardware.
Graph optimization: robust INS/USBL/DVL factor graph [83]	AMD Ryzen 7 5800U laptop; source reports Radeon Graphics at 1.90 GHz; 16 GB RAM	IMU 200 Hz; USBL 0.5 Hz; DVL 1 Hz. Optimization execution time: NR.	Source comparison: KF and HKF process only the current state and have minimal burden; the proposed fault-detection FGO is the most intensive. Peak memory: NR.	Review synthesis: possible on Nano-class hardware only with a bounded window or sparse low-rate factors; Orin-class hardware is preferable for larger graphs. Direct Jetson evidence: NR.
Learned visual SLAM: RU-SLAM [52]	Intel Core i9-9900K CPU, 3.60 GHz, 16 cores; NVIDIA RTX 2080 Ti GPU; 16 GB RAM	End-to-end runtime, FPS, and processing latency: NR.	The source uses a learned feature network with tracking, local mapping, bundle adjustment, and loop detection. Measured peak memory and per-module time: NR.	Review synthesis: the reported desktop GPU result does not establish Nano feasibility. Orin-class deployment requires model, resolution, feature count, and optimization-window profiling.
Tightly coupled visual-inertial-pressure SLAM: ORB-SLAM3-VIP [16]	CPU: NR; GPU: NR; clock: NR; RAM: NR	AQUALOC example: camera 20 Hz and pressure sensor 5 Hz; tracking runs at frame rate; scale refinement every 10 s during initialization. Execution time: NR.	Local mapping optimizes a window of keyframes and points, with joint visual, inertial, and depth residuals. Peak memory and solver time: NR.	Review synthesis: a bounded window and controlled keyframe count are required on Nano-class hardware; Orin-class hardware offers more headroom, but real-time feasibility remains unverified without target measurements.

Data source: Refs. [16,52,83,87,90]. Only source-reported numerical values are recorded; NR means Not reported. Embedded applicability is review synthesis and is not a measured Jetson Nano or Jetson Orin result.

Table S3. Detailed platform configurations for representative inland-water missions.

Typical mission	Platform and propulsion scheme	Navigation sensing and operation payloads	Power supply, communication, and control	Safety and verification priorities
Water quality and aquaculture observation in fish ponds	Small open-frame ROV or low-speed AUV, with multithruster low-speed maneuvering	IMU, depth gauge, camera, forward-looking sonar, and water quality sensors	Battery power for short voyage segments or tethered power supply, low-speed coverage, and shore-station supervision	Verify obstacle avoidance, sampling localization, and rapid recovery under interference from turbidity, fish, bubbles, and aquatic plants
River cross-section and revetment inspection	Streamlined AUV or cooperative surface and underwater platform, with flow-field compensation capability	IMU, DVL, depth gauge, forward-looking sonar, and side-scan or imaging payload	Surface relay communication, track following, dynamic obstacle avoidance, and return energy management	Verify track stability under changing flow velocity, vessels, floating objects, and DVL lock loss
Lake and reservoir mapping and ecological monitoring	Long-range AUV, with optional surface platform support for localization, communication, and recovery	INS, DVL, bathymetric sonar, water quality profiling payload, and absolute position correction information	Large-capacity battery, intermittent communication, survey transect registration, and mission replanning	Verify coverage capability under constraints of long-range drift, bottom-lock variation, communication interruption, and remaining energy margin
Inspection of dams, bridge piers, and closed facilities	ROV with multiple degrees of freedom or high-reliability AUV, with hovering, lateral motion, and propulsion redundancy	Vision, sonar, IMU, DVL, lighting, and defect detection payloads	Onboard safety closed loop, fixed-distance operation near walls, manual takeover, and emergency surfacing	Verify fault handling capability under multipath, occlusion, structural collision, localization degradation, and loss of communication

Data source: Summarized from Refs. [1–3,15,17–20,22–23,44–45,113,118,129–133,147,149–152]. The configurations are task-oriented synthesis and require revalidation under the actual water depth, flow, visibility, endurance, recovery, and failure-consequence conditions.

Table S4. Detailed hierarchical metrics, degradation modes, and evidence recommendations.

Evaluation level	Core metrics	Typical degradation/faults	Recommended evidence
Sensing and localization	ATE, RPE, consistency, availability, relocalization time	Weak texture, turbidity, multipath, DVL lock loss, time offset	Public data playback + water tank ground truth
Planning	Success rate, path cost, collision probability, replanning time, energy margin	Map changes, dynamic targets, localization covariance growth	Monte Carlo simulation + hardware-in-the-loop testing
Control	Lateral/depth error, settling time, saturation ratio, energy consumption	Flow field, parameter variation, reduced actuator efficiency, delay	High-fidelity simulation + bench/water tank tests
System and mission	Coverage rate, missed detection rate, repeat arrival error, mission success rate, maintenance labor hours	Communication interruption, computational overload, water leakage, fouling, and long-term drift	Repeated and long-term deployment in representative water areas

Data source: Synthesized from the studies and datasets discussed in Sections 6.1–6.5, including Refs. [38,50–51,214]. Evidence levels are not interchangeable, and cross-study results should be interpreted with the corresponding scenario and validation conditions.
